# Gene signatures and prognostic analyses of the Tob/BTG pituitary tumor-transforming gene (PTTG) family in clinical breast cancer patients

**DOI:** 10.7150/ijms.49652

**Published:** 2020-10-22

**Authors:** Chung-Che Wu, Titus Ime Ekanem, Nam Nhut Phan, Do Thi Thuy Loan, Sz-Ying Hou, Kuen-Haur Lee, Chih-Yang Wang

**Affiliations:** 1 Division of Neurosurgery, Department of Surgery, School of Medicine, College of Medicine, Taipei Medical University, Taipei 11031, Taiwan.; 2 PhD Program for Cancer Molecular Biology and Drug Discovery, College of Medical Science and Technology, Taipei Medical University and Academia Sinica, Taipei 11031, Taiwan.; 3 Department of Hematology, University of Uyo, Uyo 520221, Nigeria.; 4 NTT Institute of Hi-Technology, Nguyen Tat Thanh University, Ho Chi Minh City 700000, Vietnam.; 5 Graduate Institute of Cancer Biology and Drug Discovery, College of Medical Science and Technology, Taipei Medical University, Taipei 11031, Taiwan.; 6 Cancer Center, Wan Fang Hospital, Taipei Medical University, Taipei 11031, Taiwan.; 7 TMU Research Center of Cancer Translational Medicine, Taipei 11031, Taiwan.

**Keywords:** PTTG1, PTTG2, PTTG3, Breast cancer, Bioinformatics

## Abstract

Breast cancer is the most common cancer type in females, and exploring the mechanisms of disease progression is playing a crucial role in the development of potential therapeutics. Pituitary tumor-transforming gene (PTTG) family members are well documented to be involved in cell-cycle regulation and mitosis, and contribute to cancer development by their involvement in cellular transformation in several tumor types. The critical roles of PTTG family members as crucial transcription factors in diverse types of cancers are recognized, but how they regulate breast cancer development still remains mostly unknown. Meanwhile, a holistic genetic analysis exploring whether PTTG family members regulate breast cancer progression via the cell cycle as well as the energy metabolism-related network is lacking. To comprehensively understand the messenger RNA expression profiles of PTTG proteins in breast cancer, we herein conducted a high-throughput screening approach by integrating information from various databases such as Oncomine, Kaplan-Meier Plotter, Metacore, ClueGo, and CluePedia. These useful databases and tools provide expression profiles and functional analyses. The present findings revealed that PTTG1 and PTTG3 are two important genes with high expressions in breast cancer relative to normal breast cells, implying their unique roles in breast cancer progression. Results of our coexpression analysis demonstrated that PTTG family genes were positively correlated with thiamine triphosphate (TTP), deoxycytidine triphosphate (dCTP) metabolic, glycolysis, gluconeogenesis, and cell-cycle related pathways. Meanwhile, through Cytoscape analyzed indicated that in addition to the metastasis markers AURKA, AURKB, and NDC80, many of the kinesin superfamily (KIF) members including KIFC1, KIF2C, KIF4A, KIF14, KIF20A, KIF23, were also correlated with PTTG family transcript expression. Finally, we revealed that high levels of PTTG1 and PTTG3 transcription predicted poor survival, which provided useful insights into prospective research of cancer associated with the PTTG family. Therefore, these members of the PTTG family would serve as distinct and essential prognostic biomarkers in breast cancer.

## Introduction

According to the World Health Organization (WHO), 2.1 million breast cancer cases are reported annually. These cancer diseases contribute to 15% of deaths in women with cancers. In breast cancer, tumorigenesis is highly related to abnormalities in the development of the mammary epithelium, which are due to alterations in gene expressions and disruption or modifications of signal transduction pathways. Pituitary tumor-transforming gene (PTTG), GATA, signal transducer, and activator of transcription (STAT), phosphatidylinositol 3-kinase (PI3K), and NOTCH were reported to be causes of these alterations 1.

The PTTG was originally found in pituitary tumor cells of rodents in 1997 2. PTTG cellular functions are primarily associated with cell-cycle regulation and separating sister chromatids in the course of mitosis 3. Overexpression of PTTG prompts unscheduled proliferation, leading to aneuploidy and DNA instability, as well as consequent tumor development 1, 4. High PTTG expression was also found in human kidney cells, lung tumors, bladder tumors, thyroid tumors, and aggressive meningiomas, in which PTTGs are involved in cellular transformation and presumably mediate cancer onset. Furthermore, the PTTG was indicated to be an essential gene associated with cancer metastasis of primary solid tumors 5, and its upregulated expression is involved in lymph node and distant metastases 6. It is important to note the wide expression range of PTTG2 in various tumors types apart from pituitary tumors, such as testicular and liver tumors, and both ovarian tumor tissues and cell lines 7, whereas messenger (m)RNA levels of another member of the PTTG family, PTTG3, which aslo known as PTTG3P was only found to be expressed in both cell lines and tissues of ovarian tumors 8.

Upregulated expression of PTTG was also concomitantly found with upregulated levels of vascular endothelial growth factor (VEGF) and KDR 9. Upregulated mRNA expression of the PTTG was found in breast cancer 10, which was also associated with lymph node invasion and tumor recurrence 11. Cellular metabolic activity is known to be a combination of various processes of making essential cellular components and creating energy, which in turn supports other cellular processes. Perturbation of these cellular metabolic pathways was reported in cancer, in which essential enzyme activities are altered 12. In an ovarian cancer study, Wang et al. reported that PTTG is involved in regulating ovarian cell metabolism through the c-Myc pathway 13. However, associations of energy metabolism and glycolysis in breast cancer with the PTTG family remain to be determined.

In biomedical studies, high-throughput technologies play pivotal roles owing to their robustness and expeditiousness in screening for potential candidates for certain kinds of diseases. Many publicly available databases are commonly used in cancer research, such as the Gene Expression Omnibus (GEO) for raw and processed dataset analyses. Oncomine is used for querying mRNA gene expression profiles in numerous types and subtypes of cancer 14-16, and provides multiple types of analyses, including differential expression analyses of genes in tumor tissues relative to normal matched tissues. Upregulated and downregulated genes are suggested to have oncogenic or tumor-suppressive roles in cancer development 17. The PTTG was identified for its crucial role in various types of cancers, albeit, whether PTTG family members play important roles in breast cancer tumorigenesis is still an open question. Therefore, in the present study, we explored roles of PTTG members in breast cancer with public high-throughput datasets, which could initially prove distinct expressions of family members in breast cancer as well as their correlations with patient survival statuses in different breast cancer subtypes and treatment conditions. Furthermore, a functional analysis, gene ontology enrichment pathways, and network databases were explored for PTTG coexpression; these data can provide information on gene-gene interaction networks and also their signal pathways. To the best of our knowledge, this is the first systematic analysis indicating the roles of PTTG family members in breast cancer progression and tumorigenesis related to cellular metabolism.

## Materials and methods

### Oncomine analysis

To screen for mRNA expression profiles of PTTG family members in all cancers, we used the Oncomine database (www.oncomine.org). Gene symbols were input into the search box, and cancer versus normal tissue datasets were selected based on pre-defined thresholds of multiples of change (of >2-fold), *p* values (of <0.01), and gene rank percentiles (in the top 10%). A two-sample *t*-test was used to calculate significant differences between cancer tissues and normal tissues. A gene summary view was used to display datasets which satisfied the aforementioned thresholds. A color gradient was used to illustrate the decrease in the gene rank percentile. Datasets with upregulated genes of interest were colored red, whereas datasets with downregulated target genes were colored blue. Significant unique analyses showed the number of datasets that passed the threshold, and the total unique analyses meant that the entire dataset contained target genes.

### Cancer Cell Line Encyclopedia (CCLE) analysis

To further examine mRNA expression levels of PTTG family members in cancer cell lines, we used the CCLE database 18. This database contains 1457 human cancer cell lines with 136,488 unique datasets. We searched for expressions of PTTGs in 60 breast cancer cell lines with an RNA sequencing method. Expression profiles were then downloaded and plotted as a heatmap with log2-transformation of expression values with the d3heatmap package vers. 0.6.1.2 in R Studio vers. 1.2.1335 19-21.

### Metacore, ClueGO, and CluePedia for functional analyses

The METABRIC database was used to download PTTG family member coexpression profiles and the GeneGo Metacore platform was used to analyze their functions using gene ontology (GO). Processes with a correlation score of >0.7 and GO processes in the top ten were selected 22. For the GO analysis, genes with a Pearson score of >0.7 were selected for ClueGo vers. 2.3.3 and CluePedia vers. 1.3.3 23, 24. To extract the core sub-network, we first queried and downloaded a PTTG coexpression gene list from Cbioportal (https://www.cbioportal.org) with the METABRIC dataset. We chose a gene cutoff of >0.7 based on the Pearson score. Afterward, shared genes between the PTTG1, PTTG2, and PTTG3 coexpression gene lists were obtained by Venny vers 2.1 (https://bioinfogp.cnb.csic.es/tools/venny/). In total, 50 shared genes were finalized and used as input to GlueGO and CluePedia to establish a gene-gene interaction network together with their associated pathways. Both ClueGo and CluePedia analyses were performed via the Cytoscape package vers. 3.5.1. Ontology/pathway sources were updated to January 2020. GO term/Pathway selection options used default settings. An organic layout was chosen to display the best visualization, as we previously described 25-31.

### Kaplan-Meier plot of the survival analysis

Relapse-free survival (RFS) was used to study PTTG mRNA expression levels with breast cancer patient survival using the Kaplan-Meier plotter database 39. All default settings in Kaplan-Meier, such as survival curves, log-rank *p* values, and hazard ratios (HRs) with 95% confidence intervals (CIs), were retained in the plot.

## Results

### PPTG family members play crucial roles in breast cancer development

**Figure 1:**
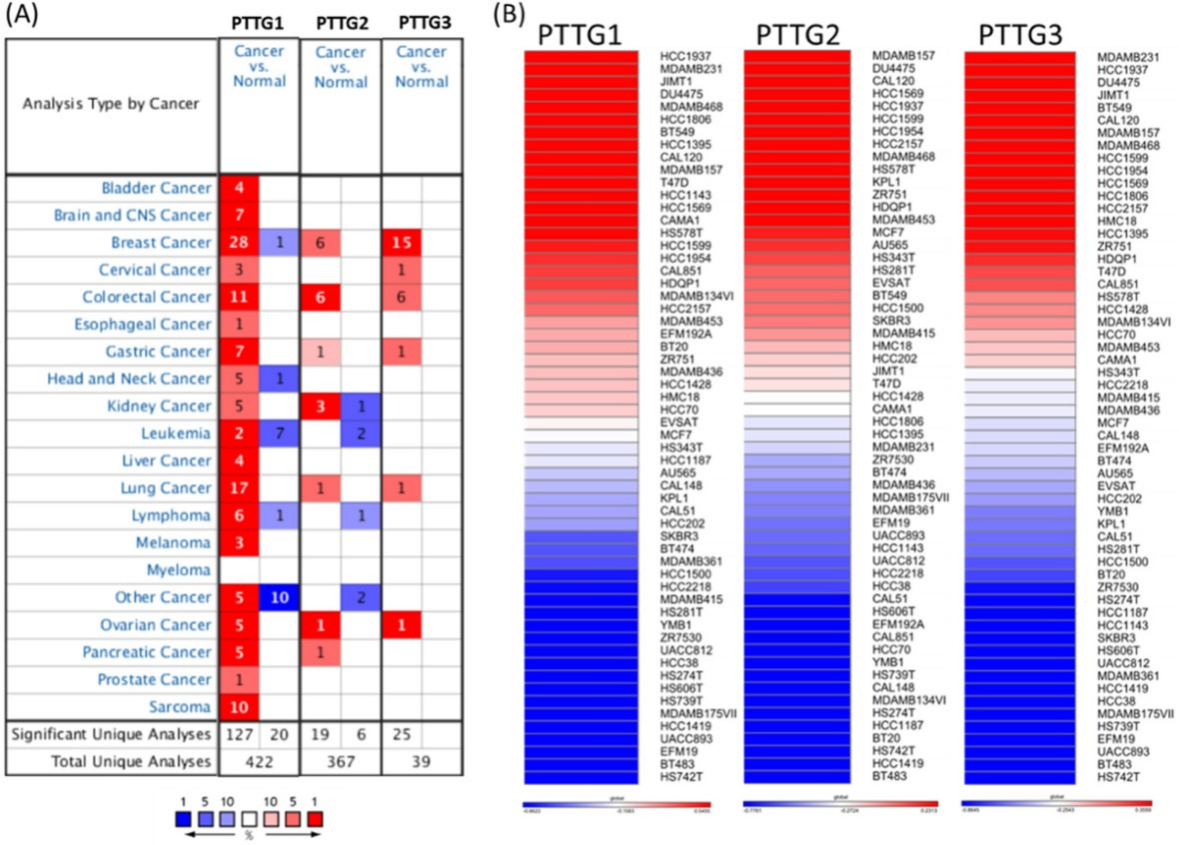
** Expressions of pituitary tumor-transforming gene (PTTG) family genes across cancer tissues.** (A) Expressions of PTTG family genes in different types of cancer compared to normal patients. The color gradient represents for decreasing in gene rank percentile. The thresholds were set to *p-value* <0.05, fold change>1.5-fold, and a gene rank percentile <10% in cancer versus normal cases. (B) PTTG expression levels with all breast cancer cell lines collected from the CCLE database were used for heatmap plot. Different breast cancer cell lines had different expression levels of PTTGs. Red indicates overexpression (up column), and blue represents under-expression (down column) in cancer cell lines.

**Figure 2:**
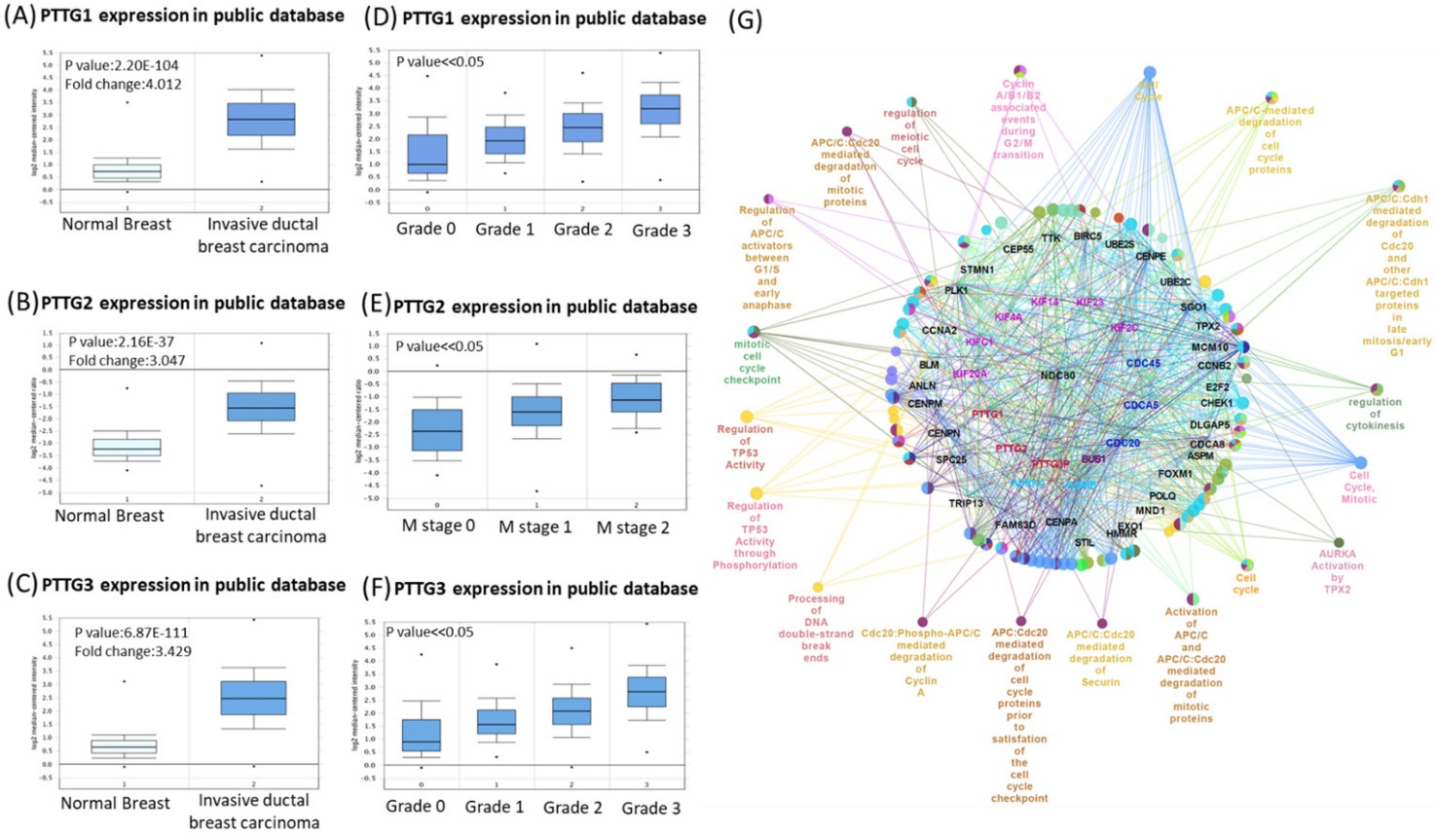
** Venn diagram of pituitary tumor-transforming gene 1 (PTTG1), PTTG2, and PTTG3 coexpression network in TCGA breast cancer databases.** (A-C) Analysis of PTTG family genes using TCGA and METABRIC databases. Comparison of expression levels of PTTG family subunits between tumors and corresponding normal tissues obtained from breast cancer patients. Box plots derived from gene expression data in TCGA breast cancer database demonstrating differential expressions of specific PTTG family members in normal and breast adenocarcinoma tissues, and *p*<0.05 was considered significant. (D-F) Analysis of PTTG family gene expressions in breast cancer patients at various stages using TCGA and METABRIC databases. Higher-stage breast cancers possessed more-elevated levels of PTTG family gene expressions than did lower-stage cancers and normal tissues. (G) Through analysis of the Cytoscape, we also found that PTTG family members had high correlations with metastasis markers, such as CDC80, AURKA, and AURKB.

Some PTTG family members were demonstrated to play crucial roles in cancer development; however, a holistic approach to exploring gene signatures in all PTTG family members is still lacking. We revealed mRNA expressions of PTTG1, PTTG2, and PTTG3 which also named as PTTG3P in 20 types of cancers (Figure 1). Among all PTTG members, PTTG1 and PTTG3 were especially distinctively highly expressed in cancers compared to normal tissues, implying their specific roles in cancer progression (Figure 1A). Next, we further explored the expression levels of PTTG family members from the CCLE database 32. The data showed that triple-negative breast cancer cell lines, such as MDA-MB-231, had high expressions of PTTG1 and PTTG3 (Figure 1B). PTTG transcripts were overexpressed in breast cancer samples relative to normal samples in TCGA and METABRIC as well as other breast cancer databases (Figure 2A-C, Table S1). In further analyses of expressions of PTTGs in breast cancer patients with different grades and stages, it was intriguing that PTTG expressions were positively correlated with tumor grades, tumor stages, and metastatic events (Figure 2D-F). Meanwhile, analysis of a published high-throughput database indicated that PTTGs were highly expressed in metastatic breast cancer 33, 34. Through a Cytoscape analysis, we also found that PTTG family members had high correlations with metastasis markers such as NDC80 35, AURKA 36, AURKB 37, and KIF2C 38 (Figure 2G).

### PPTG1 is distinctively overexpressed in breast cancer patients

**Figure 3:**
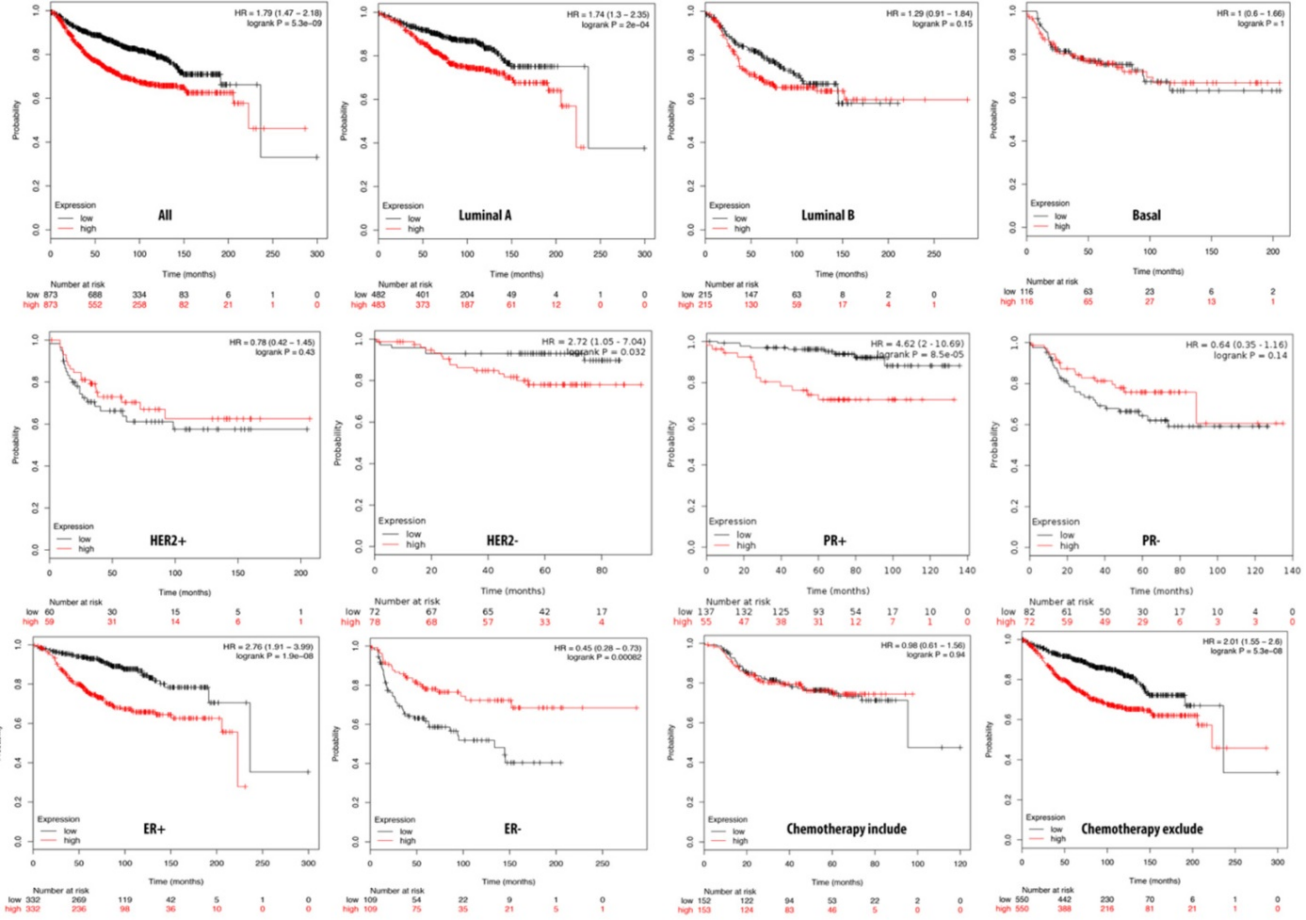
** Relationship of pituitary tumor-transforming gene 1 (PTTG1) expression and distant metastasis-free survival (DMFS) of clinical breast cancer patients (*n*=2898).** Kaplan-Meier graphs show the DMFS prognosis of breast cancer patients, based on high or low PTTG1 mRNA expression. We used the median of expression as the cutoff; therefore, patients were divided into two groups based on the overexpression and under-expression of PTTG1 mRNA. Patients with expression higher and lower than the median are shown in red and black, respectively. High expression of PTTG1 is signigicantly correlated with poor survival outcome of breast cancer patients except estrogen receptor (ER)**^-^** and progesterone receptor (PR)^-^ subtypes (total patient number=2898, *p*<0.05 was considered significant).

**Figure 4:**
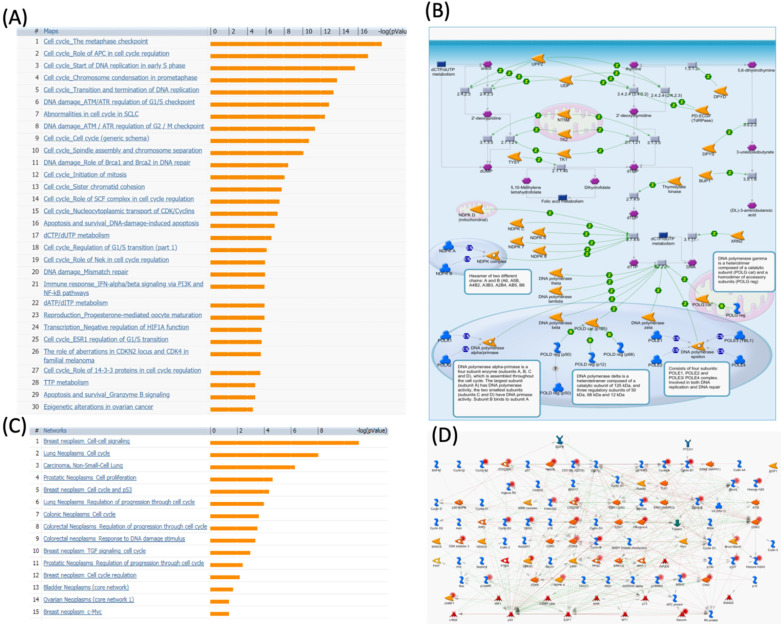
** MetaCore pathway analysis of pituitary tumor-transforming gene 1 (PTTG1) coexpressed genes in a breast cancer database.** Genes coexpressed with PTTG1 from METABRIC breast cancer patients were exported to the MetaCore pathway analysis tool to explore potential gene networks and signaling pathways impacted by the selected genes. (A) The MetaCore pathway analysis of "biology process" indicated that the cell cycle-related pathway and (B) The thiamine triphosphate (TTP) metabolic pathway were significantly associated with breast cancer development. (C) The MetaCore pathway analysis of "disease biomarker networks" indicated that the cancer-related pathway and (D) the "breast neoplasm cell-cell signaling" pathway were significantly associated with breast cancer development.

We further investigated the correlation of PTTG1 overexpression in distant metastasis-free survival (DMFS) of breast cancer patients using the Kaplan-Meier plot database. DMFS of breast cancer patients was highly correlated with PTTG1 overexpression with a hazard ratio (HR) of 1.79. In contrast, in basal, luminal A, and luminal B subtypes, overexpression of PTTG1 was highly correlated with luminal A with an HR of 1.74 (Figure 3). We also noted that patients with PTTG1 overexpression and chemotherapy treatment had a higher survival probability relative to untreated patients. Meanwhile, coexpression profiles of PTTG1 were identified from a public breast cancer database. We obtained coexpression profiles of PTTG1 with a strong cluster of the top 10% coexpressed genes across clinical breast cancer tissues from the METABRIC dataset. Afterward, GeneGo Metacore annotations of enriched biological processes indicated that PTTG1 coexpressed genes were involved in cell cycle-related molecular processes. Next, MetaCore was used to build the biological networks from the input gene set to provide the biological processes associated with each network. By uploading PTTG1 coexpression genes from the METABRIC database into the Metacore platform, we found that cell cycle-related pathways and networks such as “the metaphase checkpoint” and “TTP metabolism pathway” played essential roles in breast cancer patients (Figures 4A, B, S1, Table S2). Meanwhile, “disease biomarker networks” also indicated that “breast neoplasm cell-cell signaling” plays a vital role in breast cancer development (Figure 4C, D).

The spindle assembly checkpoint, also called the metaphase checkpoint, serves as a surveillance system playing role in delaying the start of anaphase and mitotic exit by a particular complex of proteins namely the kinetochore (Figure S1). Heterochromatin proteins HP1α and HP1γ, centromere protein B (CENP-B), and the inner centromere protein (INCENP) belong to centromeric heterochromatin 39-41. The INCENP recruits serine/threonine kinase 12 (Aurora-B) and serine/threonine kinase 13 (Aurora-C) into the kinetochore complex. Members of centromeric heterochromatin including HP1α, HP1γ, and CENP-B which are playing important role in kinetochore assembly also interact with the inner kinetochore plate. Moreover, another member of the outer kinetochore plate may inhibit CDC20. Mitotic-arrest deficient 2, yeast, homolog-like 2 (MAD2b), budding uninhibited by benzimidazoles 1 homolog (BUB1), budding uninhibited by benzimidazoles 3 homolog (BUB3), BUB1-related protein kinase (BUBR1), serine/threonine kinase 6/15 (Aurora-A), Aurora-B, and probably Aurora-C participate in recruiting these proteins to the kinetochore 42-44. Results of the pathway and network analyses suggested that PTTG1 may further regulate breast cancer development through the above spindle-assembly checkpoint and mitosis molecules.

Meanwhile, thiamine is essential for cells to maintain survival and also plays crucial roles in regulating both synaptic transmission and the central nervous system. The thiamine pathway is composed of thiamine and its derivatives namely mono- (TMP), di- (TDP), and triphosphate (TTP). TTP in its non-cofactor conformation activates maxi-chloride channel permeable property by regulating downstream protein phosphorylation. Although concentrations of TTP are low in most types of cells, comparatively high expressions exist in neuronal and excitable cells 45. Interestingly, our data showed that the “TTP metabolism pathway” was correlated with PTTG1 expression in breast cancer development (Figure 4B), which suggests that PTTG1 regulates TTP signaling in cancer progression.

### PPTG2 is distinctively overexpressed in breast cancer patients

**Figure 5:**
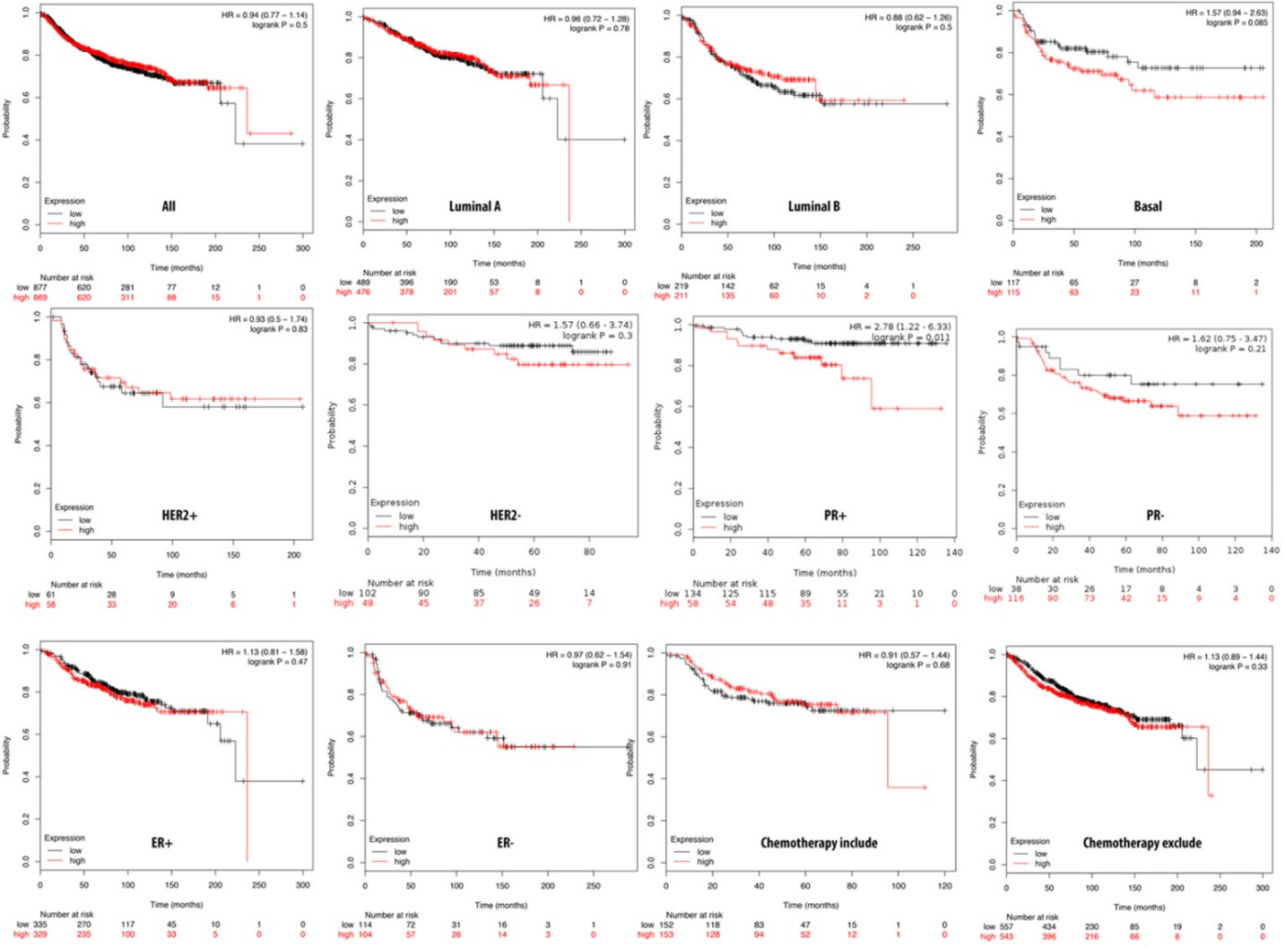
** Relationship of pituitary tumor-transforming gene 2 (PTTG2) expression and distant metastasis-free survival (DMFS) of clinical breast cancer patients (*n*=2898).** Kaplan-Meier graphs show the DMFS prognosis of breast cancer patients, based on high or low PTTG2 mRNA expression. We used the median of expression as the cutoff; therefore, patients were divided into two groups based on the overexpression and under-expression of PTTG2 mRNA. Patients with expression higher and lower than the median are shown in red and black, respectively. High expression of PTTG2 in the basal and progesterone receptor (PR) subtypes are correlated to poor survival status (total patient number=2898, *p*<0.05 considered significant).

**Figure 6:**
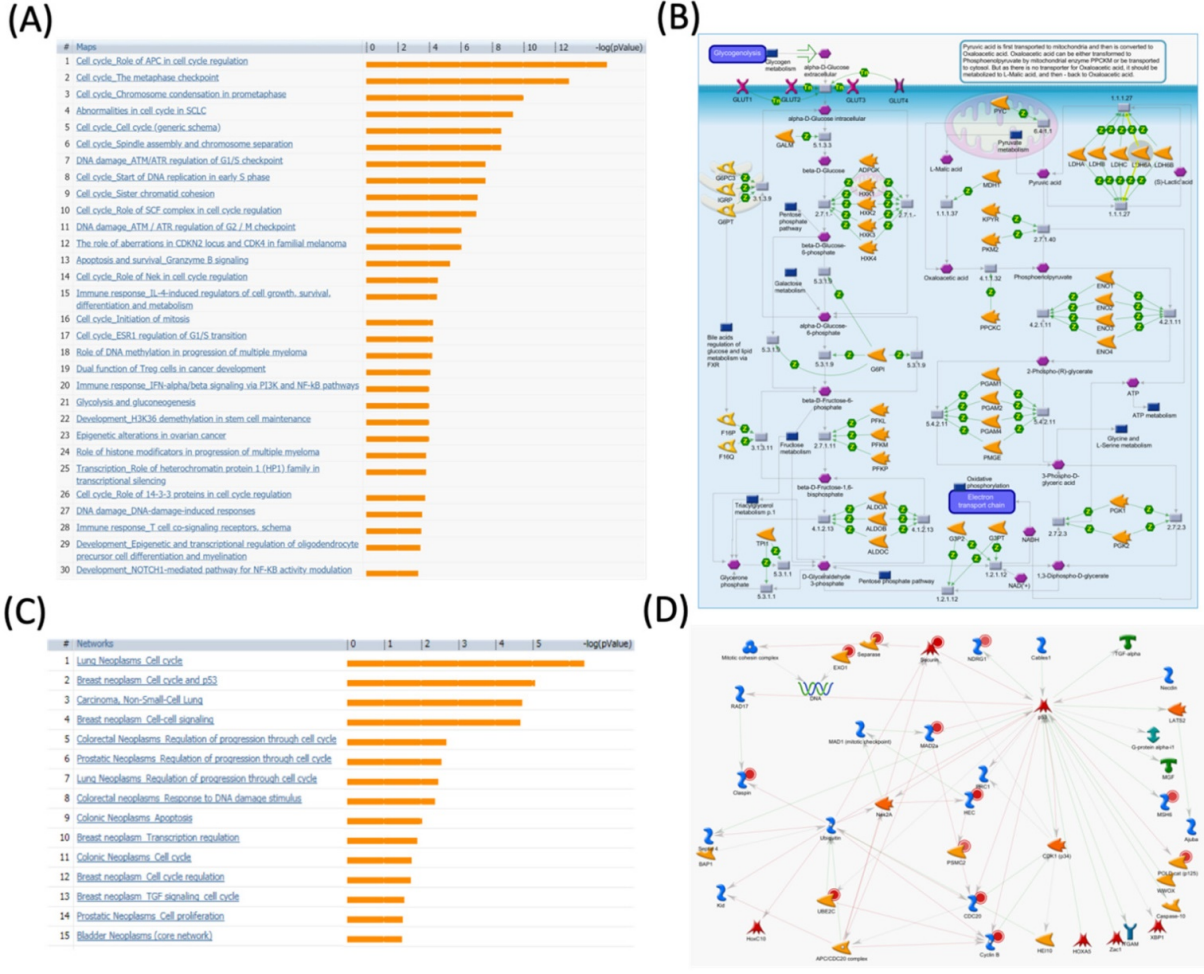
** MetaCore pathway analysis of the pituitary tumor-transforming gene 2 (PTTG2) coexpressed genes in a breast cancer database.** Genes coexpressed with PTTG2 from METABRIC breast cancer patients were exported to the MetaCore pathway analysis tool to explore potential gene networks and signaling pathways impacted by the selected genes. (A) The MetaCore pathway analysis of "biology process" indicated that the apoptosis-related pathway and (B) the "glycolysis and gluconeogenesis" pathway were significantly associated with breast cancer development. (C) The MetaCore pathway analysis of "disease biomarker networks" indicated that the cancer-related pathway and (D) the "breast neoplasm cell-cell signaling" pathway were significantly associated with breast cancer development.

The Kaplan-Meier plot database analyzed the overexpression of PTTG2 and its relationship with DMFS of breast cancer patients. Overall, DMFS of breast cancer patients was not correlated with PTTG2 overexpression. However, breast cancer patients with basal subtypes had poorer survival times when PTTG2 was overexpressed, but this was not statistically significant (HR=1.57, *p*=0.085). Being progesterone receptor (PR)-positive was significantly correlated with a shorter DMFS in patients (HR=2.78, *p*=0.01). In addition, patients who underwent chemotherapy treatment showed a better prognosis value than untreated groups with HRs of 0.91 and 1.13, respectively (Figure 5). The top 10% of genes coexpressed with PTTG2 were collected from the breast METABRIC database and uploaded to the GeneGo Metacore platform to annotate enriched biological processes. Networks related to “cell cycle role of the APC (anaphase-promoting complex) in cell cycle regulation” and “glycolysis and gluconeogenesis” were found to be the most significant pathways (Figures 6A, B, S2, Table S3). Meanwhile, the “disease biomarker networks” also indicated that “breast neoplasm cell cycle and p53” play essential roles in breast cancer development (Figure 6C, D).

The “cell cycle role of APC in cell cycle regulation” and cell division progression process are regulated by the degradation of numerous proteins (Figure S2). Ubiquitin ligases such as APC are involved in protein labeling and facilitate the degradation process by the 26S proteasome. The APC has critical roles in the cell cycle, such as inducing progression and mitosis via breaking down various cell cycle regulators, which can lead to cancer development 46. Cell division cycle 20 (CDC20) is an activator of the APC during transition of the prophase to the anaphase. In order to regulate APC activity, there are three proteins, namely CDK1, PLK1, and PKA, involved in phosphorylating the APC 47. This phosphorylation, in turn, controls the formation of CDC20 and APC activity and further regulates tumorigenesis 48. The substrate of the APC throughout the G_1_ phase is CDC20. Once CDC20 is broken down by the APC/hCDH1 resulting in APC/CDC20 inactivation, the APC/hCDH1 complex is activated.

The APC is also involved in the Aurora-A kinase degradation process during the G_1_ phase. The APC/hCDH1 conveys it. Meanwhile, overexpression of Aurora-A leads to centrosome duplication, and recent reports found that Tome-1 is required for proteolysis of some protein kinases and mitotic entry 49-51. Thus, this evidence suggests that PTTG2 may interact with the APC and Aurora-related signaling pathways to regulate cancer progression. Meanwhile, glycolysis (through the Embden-Meyerhof-Parnas pathway) and glucogenesis are two critical processes in cell energy production and glucose homeostasis. Although these two pathways are not identical, they share several steps. In glycolysis, glucose is broken down into two molecules of pyruvic acid and energy which are well-known as adenosine triphosphate (ATP) and nicotinamide adenine dinucleotide (NADH). Glycolysis is a universal pathway of glucose catabolism and is the only source of energy in some tissues and cell types. The entire pathway contains ten steps, which is typical of aerobic organisms. On the other hand, glucogenesis is the synthesis of glucose from non-carbohydrate precursors, the most important of which are lactic acid, pyruvic acid, and glycerol, as well as certain amino acids. Gluconeogenesis plays role in startvation by producing glucose. This process primarily occurs in mammal liver and an insignificant amount in renal cortex. Previous research showed that inhibition of glycolysis with citrate could serve as a cancer treatment 52, and stem cells regulated fermentative glycolysis in a breast cancer model 53. Our data showed that “glycolysis and gluconeogenesis” were correlated with PTTG2 expression in breast cancer development (Figure 6B), which suggests that PTTG2 may regulate TTP signaling in cancer progression.

### PPTG3 is distinctively overexpressed in breast cancer patients

**Figure 7:**
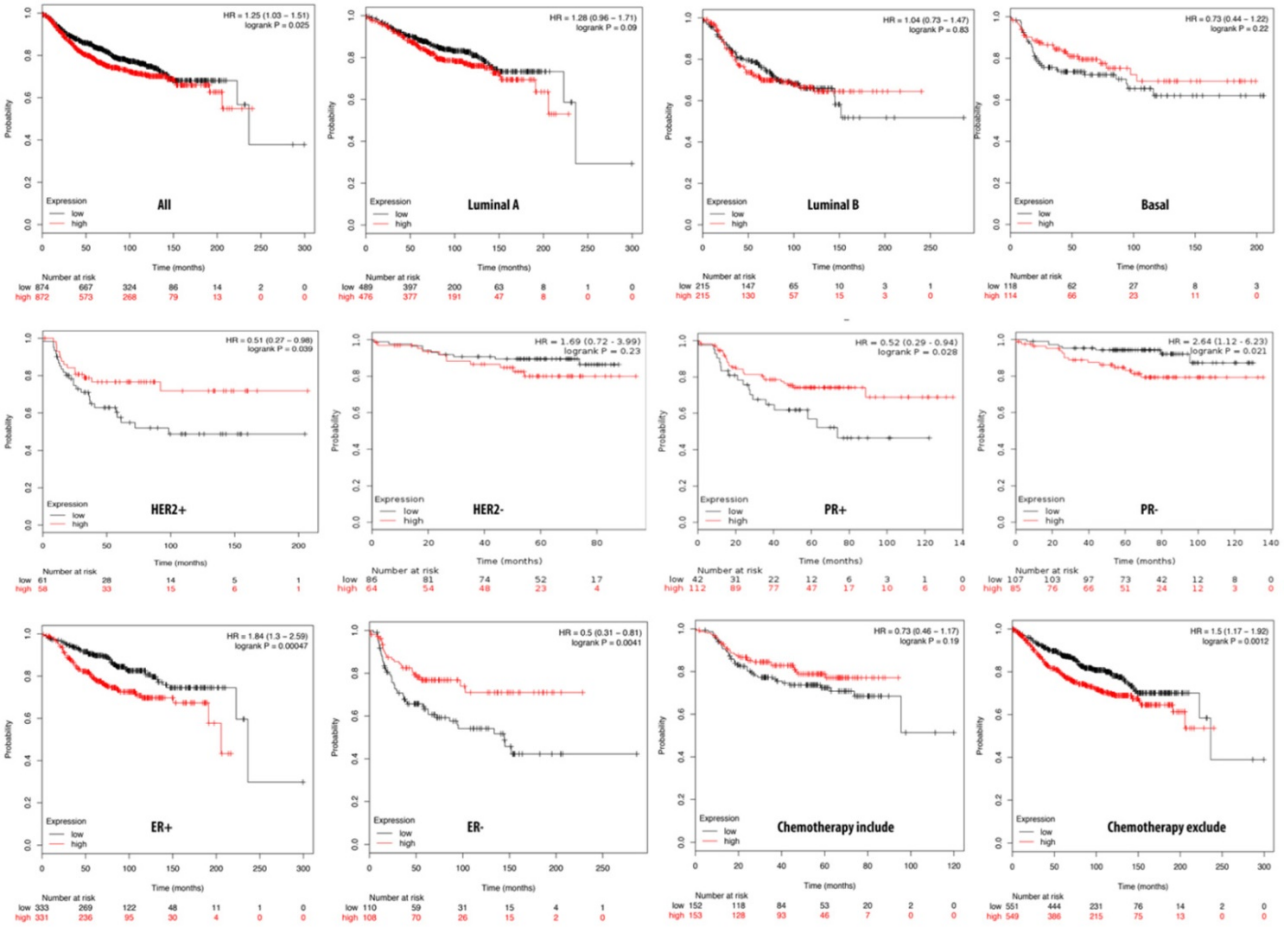
** Relationship of pituitary tumor-transforming gene 3 (PTTG3) expression and distant metastasis-free survival (DMFS) of clinical breast cancer patients (*n*=2898).** Kaplan-Meier graphs show the DMFS prognosis of breast cancer patients, based on high or low PTTG3 mRNA expression. We used the median of expression as the cutoff; therefore, patients were divided into two groups based on the overexpression and under-expression of PTTG3 mRNA. Patients with expression higher and lower than the median are shown in red and black, respectively. High PTTG3 expression was associated with poor survival in progesterone receptor (PR)^-^ and estrogen receptor (ER)^+^ subtypes and patients excluded from chemotherapy, whereas high PTTG3 expression was associated with a better survival rate (total patient number=2898, *p*<0.05 considered significant).

**Figure 8:**
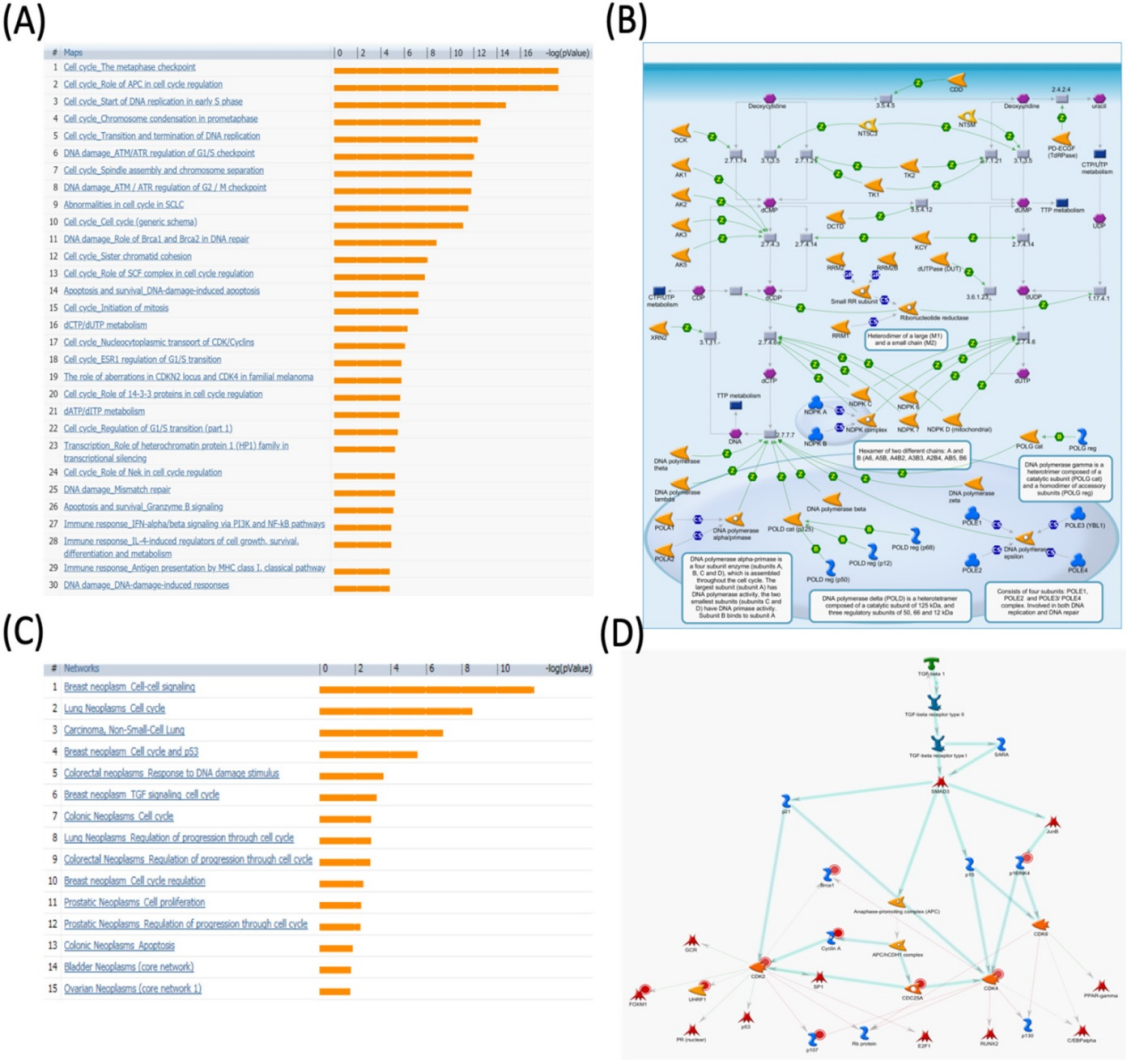
** MetaCore pathway analysis of pituitary tumor-transforming gene 3 (PTTG3) coexpressed genes in a breast cancer database.** Genes coexpressed with PTTG3 from METABRIC breast cancer patients were exported to the MetaCore pathway analysis tool to explore potential gene networks and signaling pathways impacted by the selected genes. (A) The MetaCore pathway analysis of "biology process" indicated that the estrogen (ESR)-related pathway and (B) the "dCTP/dUTP metabolism" pathway were significantly associated with breast cancer development. (C) The MetaCore pathway analysis of "disease biomarker networks" indicated that the cancer-related pathway and (D) the "breast neoplasm, TGF-β signaling, and cell cycle" pathway were significantly associated with breast cancer development.

We further investigated the correlation of the overexpression of PTTG3 with DMFS of breast cancer patients by the Kaplan-Meier plot database. The DMFS of breast cancer patients was highly correlated with overexpression of PTTG3 with an HR of 1.25. Within basal, luminal A, and luminal B subtypes, PTTG3 overexpression was highly correlated with luminal A with an HR of 1.28. A PR-positive status also had a shorter DMFS than a PR-negative status with respective HRs of 2.64 and 0.52. We also noted that patients with PTTG3 overexpression who underwent chemotherapy treatment had a higher survival probability relative to untreated patients. However, the correlation did not statistically significantly differ (*p*>0.05) (Figure 7). Coexpression profiles of PTTG3 were identified from the METABRIC database. A strong cluster of the top 10% coexpressed genes with PTTG3 was uploaded to GeneGo Metacore to annotate enriched biological processes. The “cell cycle and the metaphase checkpoint” and “dCTP/dUTP metabolism” pathways were found to be the most significant pathways for these input genes (Figures 8A, B, S3, Table S4). Meanwhile, “disease biomarker networks” also indicated that “breast neoplasm TGF-β signaling and cell cycle” plays a vital role in breast cancer development (Figure 8C, D).

The metaphase checkpoint or spindle assembly checkpoint plays a critical role in delaying the transition of the metaphase to anaphase until the completion of sister chromatid division (Figure S3). There is a so-called kinetochore which is involved in separating a cell into two new daughter cells. Free kinetochores and other members of outer kinetochores, such as MAD2B, BUB1, BUB3, and BUBR1, are inhibitors of the CDC20 homolog in *Saccharomyces cerevisiae*. Aurora-A, Aurora-B, and Aurora-C, together with CENP-A engage in assembling these CDC20 inhibitors to the kinetochore. Additionally, PLK1 also participates in the same process to recruit MAD2A and CENP-E 54-56. According to the pathway and network analyses, we believe that PTTG3 might interact with the above molecules and thereby regulate breast cancer cell division. Meanwhile, our data showed that the “dCTP/dUTP metabolism” pathway was correlated with PTTG3 expression in breast cancer development (Figure 6B), which suggests that PTTG3 regulates dCTP/dUTP signaling in cancer progression.

## Discussion

Breast cancer presents the highest prevalence and mortality among most types of cancer, especially in women. The mammary epithelium is transformed into malignant cells, which is primarily attributed to resistance to chemoradiotherapy and targeted therapy, and even distant metastasis 57, 58. Therefore, it is challenging and also necessary to illustrate the pathogenesis of breast cancer and understand how normal cells transform into cancer cells, as well as to investigate novel prognostic strategies and develop practical therapeutic approaches. Some of the members of the PTTG family present expression level in various types of cancer. PTTGs are suggested as potential therapeutic targets owing to their association with tumor progression. Previous research investigated methods of inhibiting PTTG expressions 59. Suppression of PTTG1 by small interfering (si)RNA knockdown resulted in decline transforming ability of both H1299 cells and A2780 ovarian carcinoma cells as well as attenuated tumor growth 60. Transfection of antisense PTTG1 mRNA downregulated its expression, and decreased the growth of cells of the human cervical cancer HELA-S3 cell line and increased apoptosis 61. In addition, PTTG1 was upregulated by FOXM1 in colorectal cancer, suggesting its role in migration and invasion of cancer cells 62. Our results were also consistent with previous studies, in which expression of the PTTG1 protein was found to be significantly higher in tissue samples from patients who had higher tumor grades 63, 64. Concordantly, PTTG1 overexpression results in poor prognoses of patients with myelomas, clear cell renal cell carcinoma, and adrenocortical cancer 65-67.

The roles of PTTG2 and PTTG3 in cancer are poorly studied. A previous study demonstrated that PTTG2-knockdown regulates the epithelial-to-mesenchymal transition and apoptosis 68. However, there are few studies on the functions of PTTG2 and PTTG3 in tumor biology. Therefore, our findings could provide a hint for further research exclusively in breast cancer research. PTTG family members are recognized as important transcription factors in different types of cancer. Our bioinformatics data demonstrated that among all PTTG members, PTTG1 and PTTG3 were distinctively highly expressed in breast cancer compared to normal tissues, implying their specific roles in breast cancer progression. Our coexpression analysis demonstrated that PTTG family genes were positively correlated with cell cycle-related pathways and networks. This result is consistent with a previous study which found that PTTG1 regulated cell cycle-related molecules 69, whereas to the present, this is the first report that PTTG2 and PTTG3 may also regulate breast cancer progression via cell cycle-related networks.

The survival analysis indicated that high PTTG1 expression was significantly associated with poor DMFS in all breast cancer patients, and in the luminal A and B subtypes, which implies that PTTG1 might play an oncogenic role in breast cancer. PTTG3 was significantly associated with poor DMFS in all breast cancer patients, and the luminal A subtype, which suggested that PTTG3 might also play an oncogenic role in breast cancer. Meanwhile, in the subgroup analysis, we found that elevated PTTG1 and PTTG3 expressions were correlated with a worse prognosis in patients excluded from chemotherapy, which indicated that PTTG1 and PTTG3 might play pivotal roles in chemotherapy. Therefore, PTTG1 and PTTG3 might be novel biomarkers for selecting appropriate chemotherapeutic regimens for breast cancer patients. Previous research also demonstrated that PTTG family members play crucial roles in cancer metabolism, as microRNA (miR)-186 targets PTTG1 to regulate malignant phenotypes and aerobic glycolysis 70. However, a holistic genetic analysis revealing that PTTG family members regulate breast cancer progression via cell cycle, glycolysis, and energy metabolic-related networks is lacking. Our data revealed that “TTP metabolism” was correlated with PTTG1 expression, “glycolysis/gluconeogenesis” was associated with PTTG2 expression, and the “dCTP/dUTP metabolism” pathway was correlated with PTTG3 expression in breast cancer development.

We applied a Cytoscape analysis to identify gene sets enriched in the target network of the PTTG family. Interestingly, in addition to the metastasis markers AURKA and AURKB 71, 72, many of the kinesin superfamily (KIF) members were also identified according to PTTG family transcript expression. Further mRNA expression analysis of these genes was then performed in the context of breast cancer using The Cancer Genome Atlas (TCGA) and METABRIC databases. Higher expression levels of these genes were detected compared to normal breast tissues (Figure S4). Kinesin proteins (KIFs) are highly conserved main factors in the intracellular transport system; many of them play important roles in cell division, more specifically, in mitosis and meiosis. It was suggested that dysregulation of many KIFs during the cell cycle leads to uncontrolled cell growth, therefore contributing to tumorigenesis and metastatic behaviors 73, 74. A number of kinesins were indicated to be potential therapeutic targets and predictors of clinical outcomes in various cancers, especially in breast cancer 75, 76. For instance, knocking down KIFC2 inhibited breast cancer cell growth, while introducing p53 significantly suppressed its expression 77. KIF20A was found to be involved in paclitaxel resistance and has the potential to be a predictive biomarker for breast cancer treatment 78. KIF23 overexpression was found in lung cancer cells, and its downregulation caused a cytokinesis defect and decreased the proliferation of glioma cells 79, 80, and NDC80 serves as a potential therapeutic target in lung adenocarcinoma 81. Intriguingly, our data revealed that PTTG family members regulate breast cancer progression via the cell cycle, in which KIFs and NDC80 also highly participate, highlighting the value of this family in further research.

Collectively, the present study used a holistic approach to analyze transcription profiles of PTTG family members as well as to predict their prognostic statuses in different breast cancer subtypes, in an attempt to provide useful insights in prospective research of cancer associations with the PTTG gene family; therefore, they can possibly serve as distinctive biomarkers and potential prognosticators in breast cancer.

## Supplementary Material

Supplementary figures and tables.
