# Supplementary material for: Gene signatures and prognostic analyses of the Tob/BTG pituitary tumor-transforming gene (PTTG) family in clinical breast cancer patients
Source: Int J Med Sci. 2020 Oct 22;17(18):3112–24. doi: 10.7150/ijms.49652 (PMC7646110; doi:10.7150/ijms.49652)
Supplement: Supplementary file 1 — Supplementary figures and tables. [file ijmsv17p3112s1.pdf]

Supplementary data:

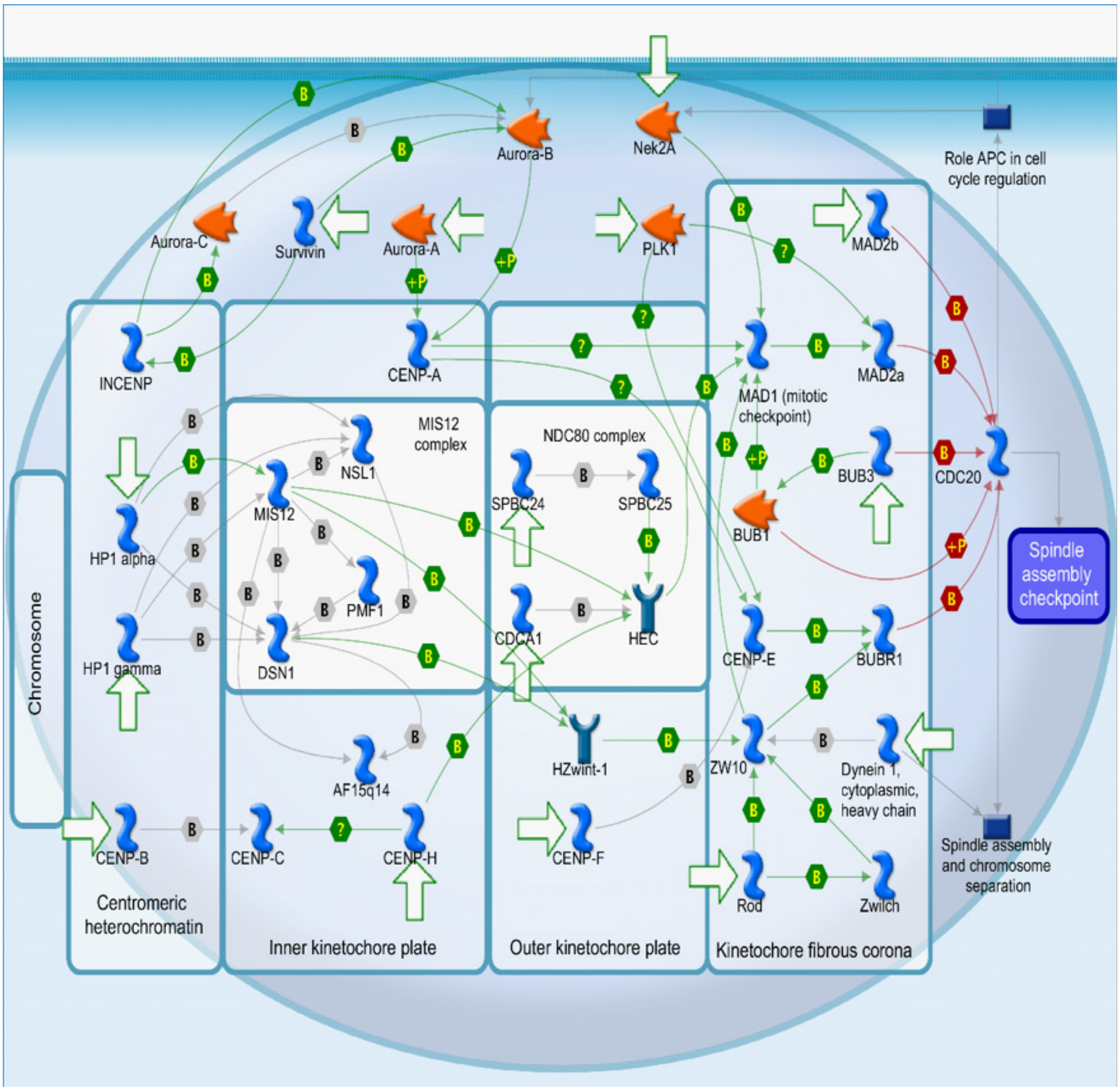

Figure S1. MetaCore pathway analysis of the coexpression gene network of pituitary tumor-transforming gene 1 (PTTG1) in breast cancer patients. Downstream pathway analyses based on the top quartile genes in Figure 4 revealed that "the metaphase checkpoint" might play an important role in breast cancer patients.

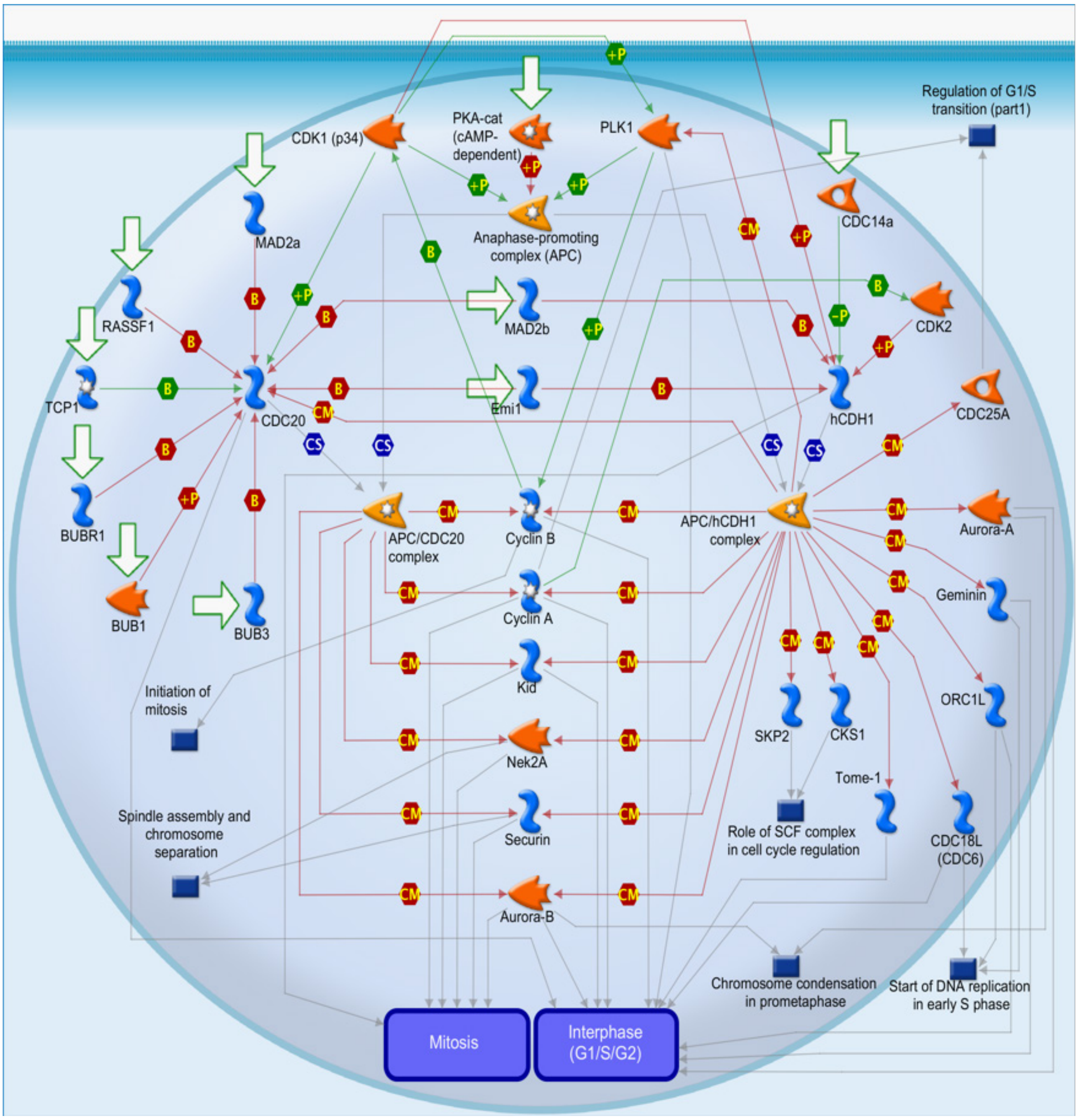

Figure S2. MetaCore pathway analysis of the coexpression gene network of pituitary tumor-transforming gene 2 (PTTG2) in breast cancer patients. Downstream pathway analyses based on the top quartile genes in Figure 6 revealed that "cell cycle role of APC in cell cycle regulation" might play an important role in breast cancer patients.

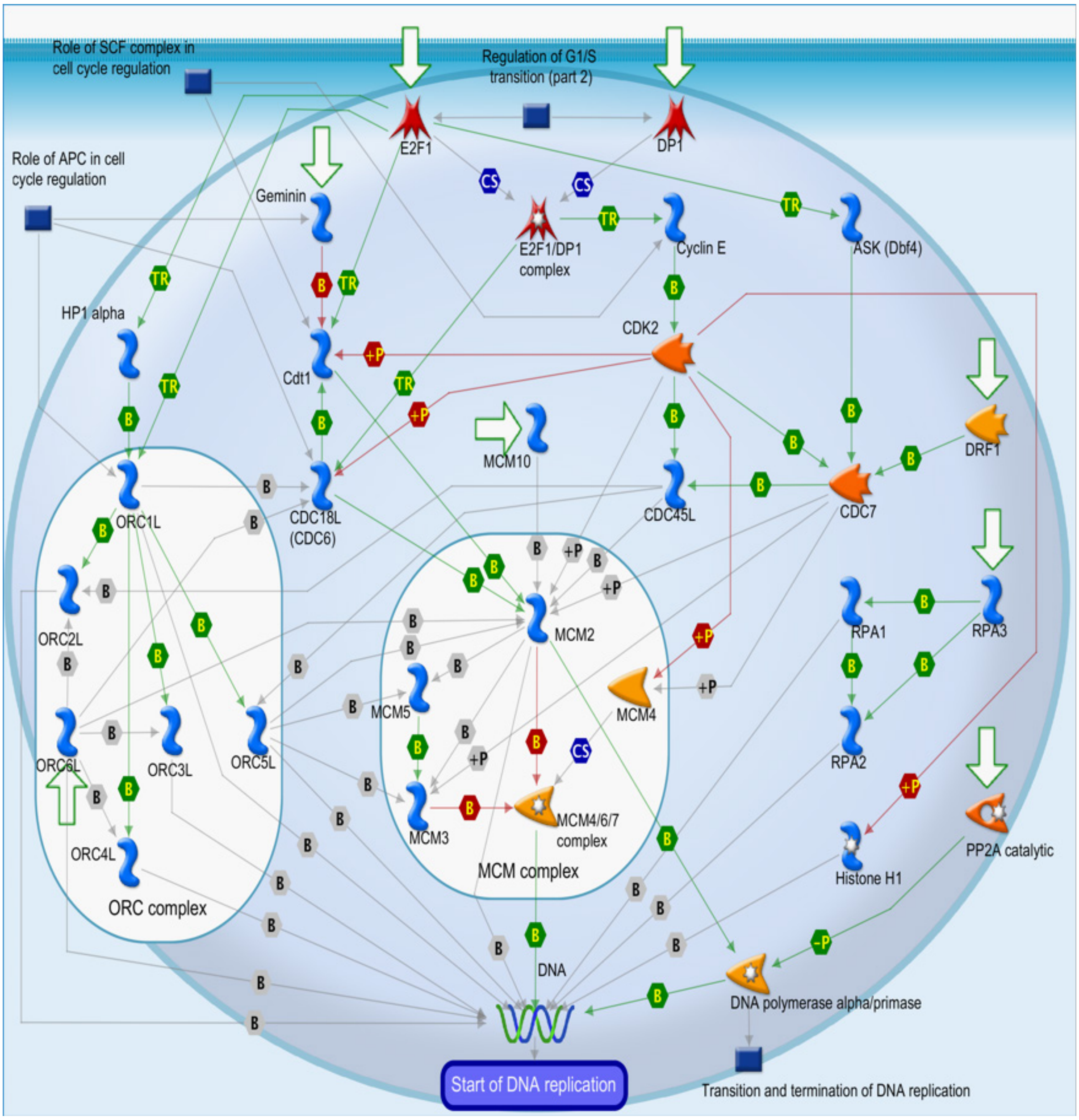

Figure S3. MetaCore pathway analysis of the coexpression gene network of pituitary tumor-transforming gene 3 (PTTG3) in breast cancer patients. Downstream pathway analyses based on the top quartile genes in Figure 8 revealed that "cell cycle and the metaphase checkpoint" might play an important role in breast cancer patients.

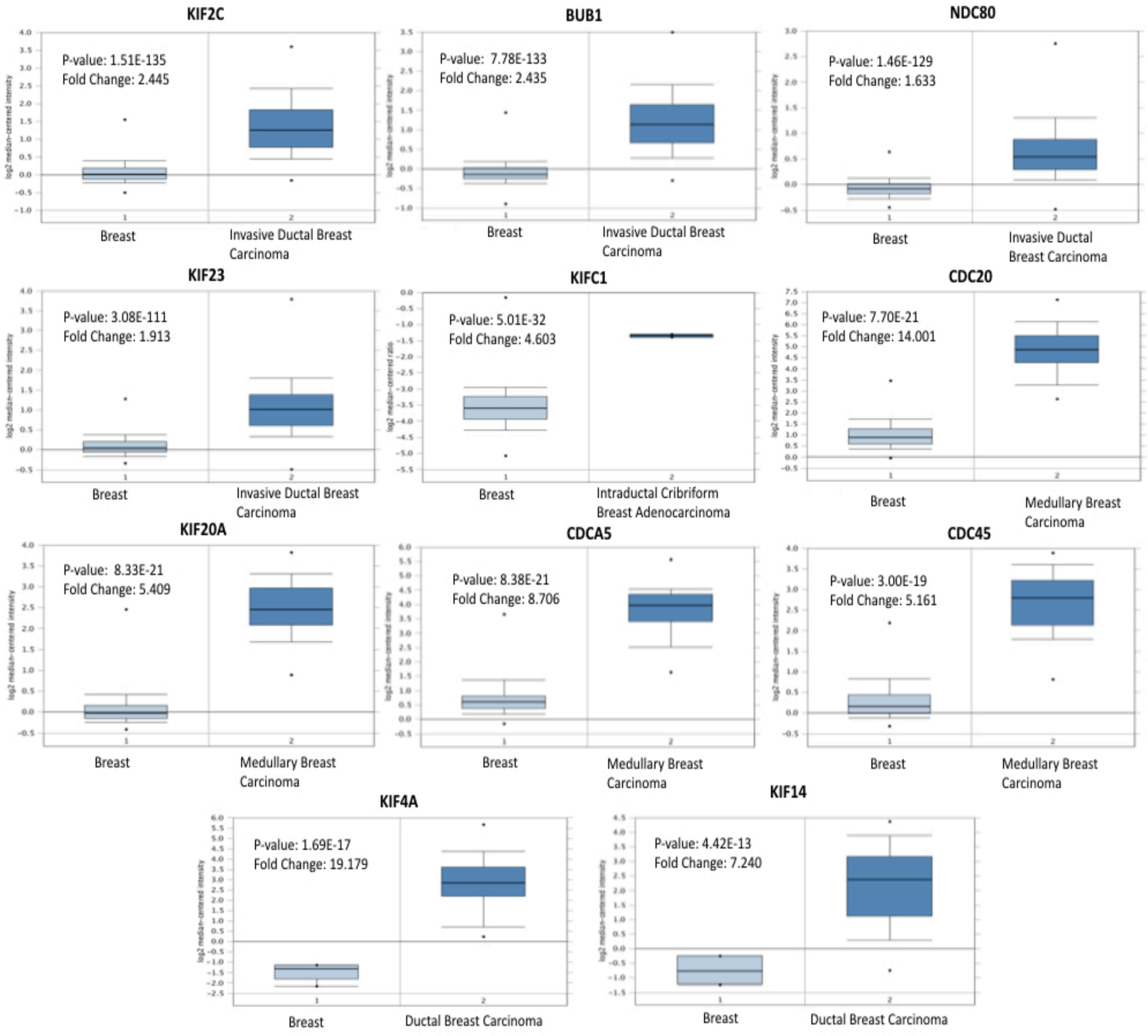

Figure S4. Meta-analysis of the PTTG family coexpressed genes in normal breast and invasive carcinoma. Further bioinformatic analysis of distinct expression patterns of genes which were found to highly correlated with PTTG family in figure 2, in normal and cancerous breast tissues.

Table S1: mRNA expression of pituitary tumor-transforming gene (PTTG) family members in different types of breast cancer patients via the Oncomine platform

Gene	Subtype	*N* (no. of cases)	*p* value (Cancer/ Normal)	*t*-Test (Cancer/ Normal)	Fold (Cancer / Normal)	% Gene Ranking	Database References
**PTTG1**	Medullary Breast Carcinoma	2136	9.60E-26	2.32E+01	10.57	1 (in top 1%)	[22]
	Invasive Breast Carcinoma	2136	7.00E-11	1.09E+01	4.79	14 (in top 1%)	[22]
	Invasive Ductal and Invasive Lobular Breast Carcinoma	2136	8.38E-42	1.89E+01	4.14	33 (in top 1%)	[22]
	Invasive Lobular Breast Carcinoma	2136	1.22E-55	2.01E+01	3.67	51 (in top 1%)	[22]
	Invasive Ductal Breast Carcinoma	2136	2.20E-104	3.99E+01	4.01	109 (in top 1%)	[22]
	Mucinous Breast Carcinoma	2136	4.62E-21	1.37E+01	3.62	110 (in top 1%)	[22]
	Breast Carcinoma	2136	5.54E-07	8.12E+00	4.78	150 (in top 1%)	[22]
	Ductal Breast Carcinoma in Situ	2136	2.43E-05	6.89E+00	3.83	194 (in top 2%)	[22]
	Tubular Breast Carcinoma	2136	1.44E-25	1.39E+01	3.15	363 (in top 2%)	[22]
	Invasive Breast Carcinoma	593	5.28E-32	1.55E+01	4.42	60 (in top 1%)	[34]
	Invasive Ductal Breast Carcinoma	593	1.27E-44	2.35E+01	5.06	111 (in top 1%)	[34]
	Invasive Lobular Breast Carcinoma	593	3.01E-16	1.04E+01	2.87	155 (in top 1%)	[34]
	Mixed Lobular and Ductal Breast Carcinoma	593	6.04E-05	6.67E+00	2.73	678 (in top 4%)	[34]
	Male Breast Carcinoma	593	7.53E-05	9.95E+00	2.65	716 (in top 4%)	[34]
	Intraductal Cribriform Breast Adenocarcinoma	593	5.45E-04	1.00E+01	3.43	1003 (in top 5%)	[34]
	Lobular Breast Carcinoma	64	4.18E-06	9.50E+00	3.27	311 (in top 3%)	[82]
	Invasive Ductal Breast Carcinoma	64	4.33E-07	9.18E+00	3.49	454 (in top 4%)	[82]
	Ductal Breast Carcinoma	85	2.00E-03	5.56E+00	3.30	312 (in top 5%)	[83]
	Ductal Breast Carcinoma	167	1.00E-03	6.33E+00	3.14	340 (in top 6%)	[84]
	Ductal Breast Carcinoma	47	1.85E-07	1.15E+01	12.29	434 (in top 3%)	[85]
	Ductal Breast Carcinoma	65	1.40E-02	4.34E+00	3.59	585 (in top 9%)	[86]
	Ductal Breast Carcinoma in Situ	63	2.20E-02	2.80E+00	3.95	701 (in top 5%)	[87]
	Invasive Ductal Breast Carcinoma	63	1.60E-02	2.56E+00	3.07	924 (in top 6%)	[87]
	Invasive Mixed Breast Carcinoma	63	2.90E-02	2.29E+00	3.08	1583 (in top 10%)	[87]
	Ductal Breast Carcinoma in Situ Stroma	66	9.91E-04	3.50E+00	2.31	849 (in top 5%)	[88]
	Invasive Ductal Breast Carcinoma Epithelia	66	4.00E-03	2.93E+00	2.32	1651 (in top 9%)	[88]
	Ductal Breast Carcinoma in	66	4.00E-03	2.89E+00	2.32	1770 (in top 10%)	[88]

	Situ Epithelia						
	Invasive Breast Carcinoma	158	2.00E-03	5.67E+00	1.72	1358 (in top 8%)	[88]
**PTTG2**	Invasive Ductal Breast Carcinoma	593	2.16E-37	1.94E+01	3.05	286 (in top 2%)	[34]
	Invasive Lobular Breast Carcinoma	593	1.92E-14	9.29E+00	2.75	297 (in top 2%)	[34]
	Invasive Breast Carcinoma	593	1.77E-23	1.21E+01	3.24	298 (in top 2%)	[34]
	Male Breast Carcinoma	593	2.23E-06	1.10E+01	2.87	462 (in top 3%)	[34]
	Intraductal Cribriform Breast Adenocarcinoma	593	2.17E-05	1.21E+01	2.58	540 (in top 3%)	[34]
	Mixed Lobular and Ductal Breast Carcinoma	593	1.36E-04	6.12E+00	2.24	915 (in top 5%)	[34]
**PTTG3P**	Medullary Breast Carcinoma	2136	1.52E-19	1.77E+01	6.16	17 (in top 1%)	[22]
	Invasive Breast Carcinoma	2136	3.63E-09	9.09E+00	3.27	60 (in top 1%)	[22]
	Invasive Ductal Carcinoma	2136	6.87E-111	3.99E+01	3.43	70 (in top 1%)	[22]
	Invasive Lobular Breast Carcinoma	2136	7.27E-40	1.60E+01	2.37	326 (in top 2%)	[22]
	Invasive Ductal and Invasive Lobular Breast Carcinoma	2136	3.69E-29	1.48E+01	2.79	327 (in top 2%)	[22]
	Mucinous Breast Carcinoma	2136	1.77E-15	1.07E+01	2.46	525 (in top 3%)	[22]
	Ductal Breast Carcinoma in Situ	2136	2.13E-04	5.29E+00	2.51	655 (in top 4%)	[22]
	Breast Carcinoma	2136	3.76E-05	5.60E+00	2.92	826 (in top 5%)	[22]
	Tubular Breast Carcinoma	2136	7.17E-20	1.17E+01	2.33	933 (in top 5%)	[22]
	Male Breast Carcinoma	593	1.14E-17	1.29E+01	2.47	67 (in top 1%)	[34]
	Invasive Ductal Breast Carcinoma	593	1.35E-45	2.41E+01	4.79	90 (in top 1%)	[34]
	Invasive Lobular Breast Carcinoma	593	1.05E-16	1.12E+01	3.33	124 (in top 1%)	[34]
	Invasive Breast Carcinoma	593	2.86E-27	1.37E+01	3.55	164 (in top 1%)	[34]
	Intraductal Cribriform Breast Adenocarcinoma	593	6.92E-06	9.94E+00	3.12	461 (in top 3%)	[34]
	Mixed Lobular and Ductal Breast Carcinoma	593	7.72E-05	6.20E+00	2.55	747 (in top 4%)	[34]

Table S2: Pathway analysis of pituitary tumor-transforming gene 1 (PTTG1)-coexpressed genes from public breast cancer databases using the MetaCore database (*p*<0.01 set as the cutoff value)

#	Map	*p* Value	Network objects from active data
**1**	Cell cycle_The metaphase checkpoint	2.3E-19	INCENP, MAD2b, SPBC25, Rod, Aurora-B, HEC, HZwint-1, Survivin, CENP-E, Nek2A, BUB1, CENP-A, HP1 alpha, Aurora-A, PLK1, CDCA1, CDC20, CENP-F, MAD2a, CENP-H, AF15q14
**2**	Cell cycle_Role of APC in cell cycle regulation	7.7E-18	MAD2b, CDC18L (CDC6), Tome-1, Cyclin A, Aurora-B, CDC25A, SKP2, Cyclin B, ORC1L, CKS1, Nek2A, BUB1, Geminin, Emi1, Aurora-A, PLK1, CDC20, MAD2a, CDK2
**3**	Cell cycle_Start of DNA replication in early S phase	2.2E-16	CDC18L (CDC6), MCM3, ORC6L, MCM4/6/7 complex, CDC7, MCM2, ORC1L, MCM5, RPA3, HP1 alpha, Geminin, DP1, MCM4, DNA polymerase alpha/primase, Cyclin E, MCM10, CDK2, CDC45L
**4**	Cell cycle_Chromosome condensation in prometaphase	1.7E-14	INCENP, CAP-H/H2, Condensin, CAP-C, Cyclin A, CNAP1, CAP-D2/D3, Aurora-B, Cyclin B, TOP2, Histone H3, CAP-G/G2, Aurora-A, CAP-E
**5**	Cell cycle_Transition and termination of DNA replication	4.4E-14	TOP2 alpha, PCNA, Cyclin A, Brca1/Bard1, Brca1, MCM2, TOP2, FEN1, POLD cat (p125), DNA ligase I, RFC complex, Bard1, DNA polymerase alpha/primase, POLD reg (p68), CDK2
**6**	DNA damage_ATM/ATR regulation of G1/S checkpoint	1.3E-13	PCNA, Chk2, I-kB, Cyclin A, Chk1, NFBD1, CDC25A, Brca1, FANCL, CDK4, BLM, FANCD2, Bard1, Cyclin E, USP1, CDK2
**7**	Abnormalities in cell cycle in SCLC	3.9E-13	PCNA, Cyclin A, p14ARF, Aurora-B, SKP2, E2F2, Histone H3, CKS1, CDK4, Cyclin B1, p16INK4, Cyclin E, E2F3, Cyclin E2, CDK2
**8**	DNA damage_ATM / ATR regulation of G2 / M checkpoint	4.4E-12	Chk2, Cyclin A, Chk1, NFBD1, Brca1, Cyclin B, Cyclin B2, FANCL, CDC25C, BLM, Cyclin B1, FANCD2, CDC25B, Kinase MYT1
**9**	Cell cycle_Cell cycle (generic schema)	1.9E-11	Cyclin A, CDC25A, Cyclin B, E2F2, CDC25C, CDK4, DP1, p107, Cyclin E, E2F3, CDC25B, CDK2

**10**	Cell cycle_Spindle assembly and chromosome separation	7.2E-11	Importin (karyopherin)-alpha, KNSL1, Aurora-B, HEC, Tubulin alpha, Cyclin B, Separase, Nek2A, TPX2, CSE1L, Aurora-A, CDC20, MAD2a, Tubulin (in microtubules)
**11**	DNA damage_Role of Brca1 and Brca2 in DNA repair	3.8E-09	PCNA, Histone H2AX, Chk2, NFBD1, Brca1/Bard1, Brca1, MSH2, FANCL, FANCD2, Rad51, MSH6, Bard1
**12**	Cell cycle_Initiation of mitosis	8.5E-09	Lamin B, Nucleolin, KNSL1, Cyclin B2, Histone H3, CDC25C, Cyclin B1, PLK1, CDC25B, FOXM1, Kinase MYT1
**13**	Cell cycle_Sister chromatid cohesion	1.8E-08	PCNA, DNA polymerase sigma, Cyclin B, RFC3, Separase, Histone H3, RFC complex, CHTF18, Rad21, DCC1
**14**	Cell cycle_Role of SCF complex in cell cycle regulation	3.4E-08	Skp2/TrCP/FBXW, Chk1, CDC25A, SKP2, CKS1, CDK4, Emi1, Cyclin E, PLK1, NEDD8, CDK2
**15**	Cell cycle_Nucleocytoplasmic transport of CDK/Cyclins	5.0E-08	Importin (karyopherin)-alpha, Cyclin A, GSK3 beta, CRM1, CDK4, Cyclin B1, Cyclin E, CDK2
**16**	Apoptosis and survival_DNA-damage-induced apoptosis	1.0E-07	Histone H2AX, Chk2, Chk1, Brca1, FANCL, BLM, FANCD2, DNA-PK
**17**	dCTP/dUTP metabolism	2.2E-07	POLE1, POLA2, POLA1, DNA polymerase theta, POLE2, POLD cat (p125), NDPK A, RRM2, TK1, DNA polymerase alpha/primase, POLD reg (p68), Small RR subunit, NT5C3, RRM1, DCK, Ribonucleotide reductase
**18**	Cell cycle_Regulation of G1/S transition (part 1)	8.0E-07	Chk2, PP2A regulatory, Cyclin A, Skp2/TrCP/FBXW, GSK3 beta, CDC25A, Brca1, CDK4, p16INK4, Cyclin E, CDK2
**19**	Cell cycle_Role of Nek in cell cycle regulation	1.2E-06	Tubulin beta, HEC, Tubulin alpha, Histone H3, Nek2A, Cyclin B1, TPX2, Aurora-A, MAD2a, Tubulin (in microtubules)
**20**	DNA damage_Mismatch repair	1.2E-06	PCNA, MutSalpha complex, DNMT1, EXO1, MSH2, Histone H3, DNA ligase I, RFC complex, MSH6, DNA-PK
**21**	Immune response_IFN-alpha/beta signaling via	1.4E-06	PCNA, 4E-BP1, I-kB, Cyclin A, GBP1, GSK3 beta, RSAD2, CDC25A,

	PI3K and NF-kB pathways		ISG15, CDK4, p16INK4, I-TAC, p107, Cyclin E, p19, DHFR, CDK2
**22**	dATP/dITP metabolism	1.4E-06	POLE1, POLA2, 8ODP, POLA1, DNA polymerase theta, POLE2, POLD cat (p125), NDPK A, RRM2, DNA polymerase alpha/primase, POLD reg (p68), Small RR subunit, ADSL, RRM1, DCK, Ribonucleotide reductase, ADA
**23**	Reproduction_Progesterone-mediated oocyte maturation	1.4E-06	PKA-reg (cAMP-dependent), GSK3 beta, CDC25C, BUB1, Cyclin B1, Aurora-A, PLK1, H-Ras, CDC20, CDC25B, Kinase MYT1
**24**	Transcription_Negative regulation of HIF1A function	2.9E-06	MCM3, p14ARF, Casein kinase I delta, GSK3 beta, HIF-prolyl hydroxylase, MCM7, ARD1, MCM2, PSMA7, MCM5, PRDX4, HSP70, Elongin C, EGLN1
**25**	Cell cycle_ESR1 regulation of G1/S transition	2.9E-06	Cyclin A, Skp2/TrCP/FBXW, CDC25A, SKP2, CRM1, CKS1, CDK4, Cyclin A2, Cyclin E, CDK2
**26**	The role of aberrations in CDKN2 locus and CDK4 in familial melanoma	3.1E-06	p14ARF, E2F3/DP1 complex, E2F2, CDK4, p16INK4, DP1, p107, E2F2/DP1 complex, E2F3
**27**	Cell cycle_Role of 14-3-3 proteins in cell cycle regulation	3.9E-06	14-3-3 gamma, Chk2, Chk1, CDC25A, CDC25C, 14-3-3 theta, 14-3-3 zeta/delta, CDC25B
**28**	TTP metabolism	1.3E-05	POLE1, POLA2, Thymidylate kinase, POLA1, UDP, DNA polymerase theta, POLE2, POLD cat (p125), NDPK A, TYSY, TK1, DNA polymerase alpha/primase, POLD reg (p68)
**29**	Apoptosis and survival_Granzyme B signaling	1.4E-05	Tubulin alpha, MUNC13-4, NOTCH1 (NEXT), Granzyme B, tBid, Caspase-3, Lamin B1, Bid, DFF40 (CAD)
**30**	Epigenetic alterations in ovarian cancer	2.3E-05	DNMT3B, DNMT1, EZH2, HRK, Aurora-B, SKP2, Brca1, Histone H3, HDAC2, CDK4, p16INK4, Aurora-A, DAPK1, CDC20
**31**	DNA damage_DNA-damage-induced responses	2.4E-05	Chk2, Chk1, NFBD1, Brca1, DNA-PK
**32**	Immune response_IL-4-induced regulators of cell growth, survival, differentiation and metabolism	2.4E-05	MCM6, Cyclin A, CDC25A, MMP-13, MCM5, CDK4, MCM4, SK4/IK1, Cyclin E, Cathepsin V, STAT1, CDK2

**33**	Cell cycle_Regulation of G1/S transition (part 2)	2.9E-05	Cyclin A, GSK3 beta, CDK4, Cyclin A2, DP1, p107, Cyclin E, CDK2
**34**	Mitogenic action of Estradiol / ESR1 (nuclear) in breast cancer	2.9E-05	CAD, CDC25A, CDK4, DNA polymerase alpha/primase, Cyclin E, SGOL2, Cyclin E2, CDK2
**35**	Immune response_Antigen presentation by MHC class I, classical pathway	3.2E-05	PSMB5, PSMB9, TAP1 (PSF1), PSME2, HSP70, Nardilysin, TAP, Calreticulin, PSMB2, IFN-gamma, TAP2 (PSF2)
**36**	Role of DNA methylation in progression of multiple myeloma	3.3E-05	DNMT1, p14ARF, NOTCH1 receptor, CDK4, SOD2, p16INK4, WNT, TDG, DAPK1, Frizzled
**37**	dGTP metabolism	3.4E-05	POLE1, POLA2, 8ODP, POLA1, DNA polymerase theta, POLE2, POLD cat (p125), NDPK A, DNA polymerase alpha/primase, POLD reg (p68), DCK, Ribonucleotide reductase
**38**	Microsatellite instability in gastric cancer	4.3E-05	PCNA, MutSalpha complex, EXO1, MSH2, RFC complex, p16INK4, MSH6
**39**	Role of histone modifiers in progression of multiple myeloma	5.1E-05	EZH2, Tubulin alpha, Histone H3, HDAC2, CDK4, N-Ras, Histone H4, Caspase-3
**40**	Transcription_Ligand-dependent activation of the ESR1/SP pathway	5.1E-05	CAD, CDC25A, TYSY, C/EBPbeta, DNA polymerase alpha/primase, Cyclin E, Cyclin E2, ADA
**41**	Cell cycle progression in Prostate Cancer	6.0E-05	4E-BP1, GSK3 beta, CDC25A, Cyclin B, CDK4, WNT, Frizzled, CDC25B, CDK2
**42**	Transcription_Role of heterochromatin protein 1 (HP1) family in transcriptional silencing	7.4E-05	DNMT1, CDC25A, Histone H3, HDAC2, Cyclin A2, HP1 alpha, Cyclin E, HP1, Histone H4
**43**	Development_NOTCH1-mediated pathway for NF-KB activity modulation	1.4E-04	NOTCH1 (NICD), SAP30, NOTCH1 receptor, Histone H3, HDAC2, NOTCH1 (NEXT), Histone H4
**44**	Immune response_IFN-gamma actions on extracellular matrix and cell differentiation	1.7E-04	GCH1, TSA-1, Casein kinase I, EZH2, GSK3 beta, WARS, HDAC2, PSME2, IFN-gamma, STAT1
**45**	ATP/ITP metabolism	1.9E-04	RRP41, RPB6, RRP40, POLR2D, RRP4, PM/SCL-75, NDPK A, RRM2, HPRT, APRT, Small RR subunit, ADSL, RRM1, RPB8, Ribonucleotide

			reductase, ADA
**46**	Immune response_T cell co-signaling receptors, schema	2.0E-04	Nectin-2, PD-1, CTLA-4, CD80, TIGIT, CD137(TNFRSF9), PVR, ICOS, LAG3, CD70(TNFSF7)
**47**	Oxidative stress_Role of ASK1 under oxidative stress	2.0E-04	14-3-3 gamma, Thioredoxin, HPK38, SOD2, PRDX1, MT-TRX, DAPK1, 14-3-3 zeta/delta, Glutaredoxin, 14-3-3
**48**	Development_H3K36 demethylation in stem cell maintenance	2.2E-04	KDM2B, EZH2, p14ARF, Histone H3, CDK4, p16INK4
**49**	Dual function of Treg cells in cancer development	2.3E-04	PD-1, CTLA-4, DNMT1, ICOS, WNT, IL-2R alpha chain, Granzyme B, IFN-gamma, STAT1
**50**	Release of pro-inflammatory mediators and elastolytic enzymes by alveolar macrophages in COPD	2.3E-04	MMP-12, HDAC2, MMP-1, IL-8, GM-CSF, IFN-gamma, STAT1

Table S3: Pathway analysis of pituitary tumor-transforming gene 2 (PTTG2)-coexpressed genes from public breast databases using the MetaCore database (*p*<0.01 set as the cutoff value)

#	**Map**	***p* Value**	**Network objects from active data**
**1**	Cell cycle_Role of APC in cell cycle regulation	4.8E-16	MAD2b, Tome-1, Cyclin A, Aurora-B, CDC25A, SKP2, Cyclin B, ORC1L, CKS1, BUB1, Geminin, Emi1, Aurora-A, PLK1, CDC20, MAD2a, Securin
**2**	Cell cycle_The metaphase checkpoint	1.3E-13	INCENP, MAD2b, SPBC25, Aurora-B, HEC, Survivin, CENP-E, BUB1, CENP-A, HP1 alpha, Aurora-A, PLK1, CDCA1, CDC20, MAD2a, AF15q14
**3**	Cell cycle_Chromosome condensation in prometaphase	9.9E-11	INCENP, CAP-H/H2, CAP-C, Cyclin A, CNAP1, CAP-D2/D3, Aurora-B, Cyclin B, Histone H3, Aurora-A, CAP-E

**4**	Abnormalities in cell cycle in SCLC	4.4E-10	PCNA, Cyclin A, p14ARF, Aurora-B, SKP2, E2F2, Histone H3, CKS1, CDK4, p16INK4, Cyclin E, E2F3
**5**	Cell cycle_Cell cycle (generic schema)	2.4E-09	Cyclin A, CDC25A, Cyclin B, E2F2, CDK4, DP1, p107, Cyclin E, E2F3, CDC25B
**6**	Cell cycle_Spindle assembly and chromosome separation	2.6E-09	Importin (karyopherin)-alpha, Aurora-B, HEC, Tubulin alpha, Cyclin B, Separase, TPX2, Aurora-A, CDC20, MAD2a, Securin, Tubulin (in microtubules)
**7**	DNA damage_ATM/ATR regulation of G1/S checkpoint	2.4E-08	PCNA, Chk2, Cyclin A, Chk1, NFBD1, CDC25A, CDK4, BLM, Cyclin E, Claspin, USP1
**8**	Cell cycle_Start of DNA replication in early S phase	2.4E-08	ORC6L, ASK (Dbf4), CDC7, ORC1L, MCM5, HP1 alpha, Geminin, DP1, Cyclin E, MCM10, CDC45L
**9**	Cell cycle_Sister chromatid cohesion	8.1E-08	PCNA, DNA polymerase sigma, Cyclin B, RFC3, Separase, Histone H3, Stromalins 1/2, DCC1, Securin
**10**	Cell cycle_Role of SCF complex in cell cycle regulation	1.0E-07	Skp2/TrCP/FBXW, Chk1, CDC25A, SKP2, CKS1, CDK4, Emi1, Cyclin E, PLK1, NEDD8
**11**	DNA damage_ATM / ATR regulation of G2 / M checkpoint	9.0E-07	Chk2, Cyclin A, Chk1, NFBD1, Cyclin B, Cyclin B2, BLM, Claspin, CDC25B
**12**	The role of aberrations in CDKN2 locus and CDK4 in familial melanoma	9.0E-07	p14ARF, E2F3/DP1 complex, E2F2, CDK4, p16INK4, DP1, p107, E2F2/DP1 complex, E2F3
**13**	Apoptosis and survival_Granzyme B signaling	4.2E-06	Caspase-2, Tubulin alpha, NOTCH1 (NEXT), Granzyme B, tBid, Caspase-3, Lamin B1, Bid, DFF40 (CAD)
**14**	Cell cycle_Role of Nek in cell cycle regulation	2.9E-05	Tubulin beta, HEC, Tubulin alpha, Histone H3, TPX2, Aurora-A, MAD2a, Tubulin (in microtubules)
**15**	Immune response_IL-4-induced regulators of cell growth, survival, differentiation and metabolism	3.2E-05	MCM6, PERC, Cyclin A, CDC25A, MMP-13, MCM5, CDK4, SK4/IK1, Cyclin E, Cathepsin V, STAT1

**16**	Cell cycle_Initiation of mitosis	5.6E-05	Lamin B, Nucleolin, Cyclin B2, Histone H3, PLK1, CDC25B, FOXM1
**17**	Cell cycle_ESR1 regulation of G1/S transition	5.9E-05	Cyclin A, Skp2/TrCP/FBXW, CDC25A, SKP2, CKS1, CDK4, Cyclin A2, Cyclin E
**18**	Role of DNA methylation in progression of multiple myeloma	6.3E-05	DNMT1, p14ARF, NOTCH1 receptor, CDK4, SOD2, p16INK4, WNT, DAPK1, Frizzled
**19**	Dual function of Treg cells in cancer development	7.6E-05	PD-1, CTLA-4, DNMT1, ICOS, WNT, IL-2R alpha chain, Granzyme B, IFN-gamma, STAT1
**20**	Immune response_IFN-alpha/beta signaling via PI3K and NF-kB pathways	9.6E-05	PCNA, 4E-BP1, Cyclin A, GBP1, CDC25A, CDK4, p16INK4, I-TAC, p107, Cyclin E, p19, DHFR, eIF4A
**21**	Glycolysis and gluconeogenesis	9.6E-05	G3P2, TPI1, GLUT1, ENO1, PGAM1, LDHB, HXK3, ALDOC, PFKP, MDH1, PGK1, PGAM4, GPI
**22**	Development_H3K36 demethylation in stem cell maintenance	9.9E-05	KDM2B, EZH2, p14ARF, Histone H3, CDK4, p16INK4
**23**	Epigenetic alterations in ovarian cancer	1.0E-04	DNMT1, EZH2, HRK, Aurora-B, SKP2, Histone H3, HDAC2, CDK4, p16INK4, Aurora-A, DAPK1, CDC20
**24**	Role of histone modifiers in progression of multiple myeloma	1.5E-04	EZH2, Tubulin alpha, Histone H3, HDAC2, CDK4, N-Ras, Caspase-3
**25**	Transcription_Role of heterochromatin protein 1 (HP1) family in transcriptional silencing	1.6E-04	DNMT1, CDC25A, Histone H3, HDAC2, Cyclin A2, HP1 alpha, Cyclin E, HP1
**26**	Cell cycle_Role of 14-3-3 proteins in cell cycle regulation	1.8E-04	14-3-3 gamma, Chk2, Chk1, CDC25A, 14-3-3 theta, CDC25B
**27**	DNA damage_DNA-damage-induced responses	2.8E-04	Chk2, Chk1, NFBD1, DNA-PK
**28**	Immune response_T cell co-signaling receptors, schema	3.2E-04	Nectin-2, PD-1, CTLA-4, TIGIT, PVR, ICOS, CD30(TNFRSF8), LAG3, PD-L1

**29**	Development_Epigenetic and transcriptional regulation of oligodendrocyte precursor cell differentiation and myelination	3.4E-04	NOTCH1 (NICD), SOX11, UGT8, NOTCH1 receptor, PPAR-beta(delta), HDAC2, Frizzled
**30**	Development_NOTCH1-mediated pathway for NF-KB activity modulation	4.8E-04	NOTCH1 (NICD), SAP30, NOTCH1 receptor, Histone H3, HDAC2, NOTCH1 (NEXT)
**31**	Immune response_Induction of apoptosis and inhibition of proliferation mediated by IFN-gamma	5.2E-04	IDO1, GBP1, WARS, CDK4, Cyclin E, Caspase-3, IFN-gamma, STAT1
**32**	Rb proteins signaling in multiple myeloma	5.8E-04	p18, CDK4, p16INK4, p107, Cyclin E
**33**	Cell cycle_Regulation of G1/S transition (part 1)	7.0E-04	Chk2, Cyclin A, Skp2/TrCP/FBXW, CDC25A, CDK4, p16INK4, Cyclin E
**34**	Neutrophil chemotaxis in asthma	7.0E-04	GRO-2, FPRL1, G-protein beta/gamma, GRO-1, HSP70, Tissue kallikreins, CCL7
**35**	Cell cycle_Regulation of G1/S transition (part 2)	7.3E-04	Cyclin A, CDK4, Cyclin A2, DP1, p107, Cyclin E
**36**	Release of pro-inflammatory mediators and elastolytic enzymes by alveolar macrophages in COPD	7.3E-04	MMP-12, HDAC2, MMP-1, GM-CSF, IFN-gamma, STAT1
**37**	Cell cycle progression in Prostate Cancer	8.3E-04	4E-BP1, CDC25A, Cyclin B, CDK4, WNT, Frizzled, CDC25B
**38**	Inter-cellular relations in COPD (general schema)	1.1E-03	GRO-1, HDAC2, I-TAC, Granzyme B, GM-CSF, IFN-gamma
**39**	Transcription_Ligand-dependent activation of the ESR1/SP pathway	1.1E-03	CAD, CDC25A, TYSY, C/EBPbeta, Cyclin E, ADA
**40**	DNA damage_Role of Brca1 and Brca2 in DNA repair	1.1E-03	PCNA, Chk2, NFBD1, Brca2, Rad51, MSH6
**41**	Role of Bregs in attenuation of T and NK cells mediated anti-tumor immune responses	1.1E-03	PD-1, CTLA-4, IL-2R alpha chain, Granzyme B, LAG3, IFN-gamma, PD-L1
**42**	Putative pathways of MHC class I-dependent	1.2E-03	TAP1 (PSF1), PIRB, TAP, IFN-gamma, TAP2 (PSF2)

	postsynaptic long-term depression in major depressive disorder		
**43**	Inhibition of tumor suppressive pathways in pancreatic cancer	1.2E-03	p14ARF, Brca2, CDK4, p16INK4, Rad51
**44**	Basophil migration in asthma	1.5E-03	FPRL1, G-protein beta/gamma, CCL13, CCL18, FPRL2, CCL8, GM-CSF, CCL7
**45**	Oxidative stress_Role of ASK1 under oxidative stress	1.5E-03	14-3-3 gamma, Thioredoxin, HPK38, SOD2, MT-TRX, DAPK1, Glutaredoxin, 14-3-3
**46**	Immune response_Antimicrobial actions of IFN-gamma	1.5E-03	Esrra, PERC, IDO1, GBP1, C/EBPbeta, IFN-gamma, STAT1
**47**	Development_Notch Signaling Pathway	1.5E-03	NOTCH1 (NICD), SAP30, NOTCH1 receptor, Histone H3, HDAC2, NOTCH1 (NEXT), NOTCH1 precursor
**48**	DNA damage_Mismatch repair	1.5E-03	PCNA, DNMT1, EXO1, Histone H3, MSH6, DNA-PK
**49**	Apoptosis and survival_Regulation of Apoptosis by Mitochondrial Proteins	1.8E-03	BFL1, HRK, Bak, ANT, tBid, Bid
**50**	Cell cycle_Nucleocytoplasmic transport of CDK/Cyclins	1.9E-03	Importin (karyopherin)-alpha, Cyclin A, CDK4, Cyclin E

Table S4: Pathway analysis of pituitary tumor-transforming gene 3 (PTTG3P)-coexpressed genes from public breast databases using the MetaCore database (p<0.01 set as the cutoff value)

#	**Map**	***p* Value**	**Network objects from active data**
**1**	Cell cycle_The metaphase checkpoint	4.59E-20	INCENP, MAD2b, SPBC25, Rod, Aurora-B, HEC, HZwint-1, Survivin, CENP-E, Nek2A, BUB1, CENP-A, HP1 alpha, Aurora-A, PLK1,

			CDCA1, CDC20, CENP-F, MAD2a, CENP-H, AF15q14
**2**	Cell cycle_Role of APC in cell cycle regulation	5.05E-20	MAD2b, CDC18L (CDC6), Tome-1, Cyclin A, Aurora-B, CDC25A, SKP2, Cyclin B, ORC1L, CKS1, Nek2A, BUB1, Geminin, Emi1, Aurora-A, PLK1, CDC20, MAD2a, Securin, CDK2
**3**	Cell cycle_Start of DNA replication in early S phase	1.54E-15	CDC18L (CDC6), MCM3, ORC6L, MCM4/6/7 complex, CDC7, MCM2, ORC1L, MCM5, HP1 alpha, Geminin, DP1, MCM4, DNA polymerase alpha/primase, Cyclin E, MCM10, CDK2, CDC45L
**4**	Cell cycle_Chromosome condensation in prometaphase	2.35E-13	INCENP, CAP-H/H2, Condensin, CAP-C, Cyclin A, CNAP1, CAP-D2/D3, Aurora-B, Cyclin B, TOP2, CAP-G/G2, Aurora-A, CAP-E
**5**	Cell cycle_Transition and termination of DNA replication	4E-13	TOP2 alpha, PCNA, Cyclin A, Brca1/Bard1, Brca1, MCM2, TOP2, FEN1, POLD cat (p125), DNA ligase I, RFC complex, Bard1, DNA polymerase alpha/primase, CDK2
**6**	DNA damage_ATM/ATR regulation of G1/S checkpoint	8.06E-13	PCNA, Chk2, Cyclin A, Chk1, NFBD1, CDC25A, Brca1, FANCL, CDK4, BLM, FANCD2, Bard1, Cyclin E, USP1, CDK2
**7**	Cell cycle_Spindle assembly and chromosome separation	1.42E-12	Importin (karyopherin)-alpha, KNSL1, Aurora-B, HEC, Tubulin alpha, Cyclin B, Separase, Nek2A, TPX2, CSE1L, Aurora-A, CDC20, MAD2a, Securin, Tubulin (in microtubules)
**8**	DNA damage_ATM / ATR regulation of G2 / M checkpoint	1.53E-12	Chk2, Cyclin A, Chk1, NFBD1, Brca1, Cyclin B, Cyclin B2, FANCL, CDC25C, BLM, Cyclin B1, FANCD2, CDC25B, Kinase MYT1
**9**	Abnormalities in cell cycle in SCLC	2.85E-12	PCNA, Cyclin A, p14ARF, Aurora-B, SKP2, E2F2, CKS1, CDK4, Cyclin B1, p16INK4, Cyclin E, E2F3, Cyclin E2, CDK2
**10**	Cell cycle_Cell cycle (generic schema)	7.72E-12	Cyclin A, CDC25A, Cyclin B, E2F2, CDC25C, CDK4, DP1, p107, Cyclin E, E2F3, CDC25B, CDK2
**11**	DNA damage_Role of Brca1 and Brca2 in DNA repair	1.59E-09	PCNA, Histone H2AX, Chk2, NFBD1, Brca1/Bard1, Brca1, MSH2, FANCL, FANCD2, Rad51, MSH6, Bard1

**12**	Cell cycle_Sister chromatid cohesion	8.44E-09	PCNA, DNA polymerase sigma, Cyclin B, RFC3, Separase, RFC complex, CHTF18, Rad21, DCC1, Securin
**13**	Cell cycle_Role of SCF complex in cell cycle regulation	1.51E-08	Skp2/TrCP/FBXW, Chk1, CDC25A, SKP2, CKS1, CDK4, Emi1, Cyclin E, PLK1, NEDD8, CDK2
**14**	Apoptosis and survival_DNA-damage-induced apoptosis	5.67E-08	Histone H2AX, Chk2, Chk1, Brca1, FANCL, BLM, FANCD2, DNA-PK
**15**	Cell cycle_Initiation of mitosis	5.92E-08	Lamin B, Nucleolin, KNSL1, Cyclin B2, CDC25C, Cyclin B1, PLK1, CDC25B, FOXM1, Kinase MYT1
**16**	dCTP/dUTP metabolism	4.83E-07	POLE1, POLA2, POLA1, DNA polymerase theta, POLE2, POLD cat (p125), NDPK A, RRM2, TK1, DNA polymerase alpha/primase, Small RR subunit, NT5C3, RRM1, DCK, Ribonucleotide reductase
**17**	Cell cycle_Nucleocytoplasmic transport of CDK/Cyclins	7.12E-07	Importin (karyopherin)-alpha, Cyclin A, CRM1, CDK4, Cyclin B1, Cyclin E, CDK2
**18**	Cell cycle_ESR1 regulation of G1/S transition	1.44E-06	Cyclin A, Skp2/TrCP/FBXW, CDC25A, SKP2, CRM1, CKS1, CDK4, Cyclin A2, Cyclin E, CDK2
**19**	The role of aberrations in CDKN2 locus and CDK4 in familial melanoma	1.62E-06	p14ARF, E2F3/DP1 complex, E2F2, CDK4, p16INK4, DP1, p107, E2F2/DP1 complex, E2F3
**20**	Cell cycle_Role of 14-3-3 proteins in cell cycle regulation	2.15E-06	14-3-3 gamma, Chk2, Chk1, CDC25A, CDC25C, 14-3-3 theta, 14-3-3 zeta/delta, CDC25B
**21**	dATP/dITP metabolism	2.37E-06	POLE1, POLA2, 8ODP, POLA1, DNA polymerase theta, POLE2, POLD cat (p125), NDPK A, RRM2, DNA polymerase alpha/primase, Small RR subunit, ADSL, RRM1, DCK, Ribonucleotide reductase, ADA
**22**	Cell cycle_Regulation of G1/S transition (part 1)	3.3E-06	Chk2, PP2A regulatory, Cyclin A, Skp2/TrCP/FBXW, CDC25A, Brca1, CDK4, p16INK4, Cyclin E, CDK2
**23**	Transcription_Role of heterochromatin protein 1	5.47E-06	Mi-2, DNMT1, CDC25A, HDAC2, Cyclin A2, HP1 alpha, Cyclin E, HP1,

	(HP1) family in transcriptional silencing		Histone H4, Mi-2 alpha
**24**	Cell cycle_Role of Nek in cell cycle regulation	5.64E-06	Tubulin beta, HEC, Tubulin alpha, Nek2A, Cyclin B1, TPX2, Aurora-A, MAD2a, Tubulin (in microtubules)
**25**	DNA damage_Mismatch repair	5.64E-06	PCNA, MutSalpha complex, DNMT1, EXO1, MSH2, DNA ligase I, RFC complex, MSH6, DNA-PK
**26**	Apoptosis and survival_Granzyme B signaling	7.46E-06	Tubulin alpha, MUNC13-4, NOTCH1 (NEXT), Granzyme B, tBid, Caspase-3, Lamin B1, Bid, DFF40 (CAD)
**27**	Immune response_IFN-alpha/beta signaling via PI3K and NF-kB pathways	1.11E-05	PCNA, 4E-BP1, Cyclin A, GBP1, RSAD2, CDC25A, ISG15, CDK4, p16INK4, I-TAC, p107, Cyclin E, p19, DHFR, CDK2
**28**	Immune response_IL-4-induced regulators of cell growth, survival, differentiation and metabolism	1.12E-05	MCM6, Cyclin A, CDC25A, MMP-13, MCM5, CDK4, MCM4, SK4/IK1, Cyclin E, Cathepsin V, STAT1, CDK2
**29**	Immune response_Antigen presentation by MHC class I, classical pathway	1.56E-05	PSMB5, PSMB9, TAP1 (PSF1), PSME2, HSP70, Nardilysin, TAP, Calreticulin, PSMB2, IFN-gamma, TAP2 (PSF2)
**30**	DNA damage_DNA-damage-induced responses	1.61E-05	Chk2, Chk1, NFBD1, Brca1, DNA-PK
**31**	Mitogenic action of Estradiol / ESR1 (nuclear) in breast cancer	1.66E-05	CAD, CDC25A, CDK4, DNA polymerase alpha/primase, Cyclin E, SGOL2, Cyclin E2, CDK2
**32**	Microsatellite instability in gastric cancer	2.61E-05	PCNA, MutSalpha complex, EXO1, MSH2, RFC complex, p16INK4, MSH6
**33**	Transcription_Ligand-dependent activation of the ESR1/SP pathway	2.9E-05	CAD, CDC25A, TYSY, C/EBPbeta, DNA polymerase alpha/primase, Cyclin E, Cyclin E2, ADA
**34**	TTP metabolism	2.98E-05	POLE1, POLA2, Thymidylate kinase, POLA1, UDP, DNA polymerase theta, POLE2, POLD cat (p125), NDPK A, TYSY, TK1, DNA polymerase alpha/primase
**35**	Regulation of degradation of deltaF508-CFTR in CF	3.25E-05	SEC61 complex, UFD1, Hdj-2, Csp, Sti1, HSP70, SAE1, Derlin1, UCHL1

**36**	Reproduction_Progesterone-mediated oocyte maturation	4.04E-05	CDC25C, BUB1, Cyclin B1, Aurora-A, PLK1, H-Ras, CDC20, CDC25B, Kinase MYT1
**37**	Epigenetic alterations in ovarian cancer	4.56E-05	DNMT3B, DNMT1, EZH2, HRK, Aurora-B, SKP2, Brca1, HDAC2, CDK4, p16INK4, Aurora-A, DAPK1, CDC20
**38**	ATP/ITP metabolism	7.69E-05	RRP41, RPB6, POLR2D, RRP4, PM/SCL-75, NDPK A, RRM2, RRP46, HPRT, APRT, Small RR subunit, ADSL, RRM1, RPB8, Ribonucleotide reductase, ADA
**39**	dGTP metabolism	8.19E-05	POLE1, POLA2, 8ODP, POLA1, DNA polymerase theta, POLE2, POLD cat (p125), NDPK A, DNA polymerase alpha/primase, DCK, Ribonucleotide reductase
**40**	Oxidative stress_Role of ASK1 under oxidative stress	1.05E-04	14-3-3 gamma, Thioredoxin, HPK38, SOD2, PRDX1, MT-TRX, DAPK1, 14-3-3 zeta/delta, Glutaredoxin, 14-3-3
**41**	Cell cycle_Regulation of G1/S transition (part 2)	1.45E-04	Cyclin A, CDK4, Cyclin A2, DP1, p107, Cyclin E, CDK2
**42**	Release of pro-inflammatory mediators and elastolytic enzymes by alveolar macrophages in COPD	1.45E-04	MMP-12, IP10, HDAC2, MMP-1, GM-CSF, IFN-gamma, STAT1
**43**	IL-6 signaling in colorectal cancer	1.47E-04	Ku70, DNMT1, Cyclin B, Survivin, Cyclin B1, HSP70, Cyclin E, Clusterin
**44**	Immune response_Induction of apoptosis and inhibition of proliferation mediated by IFN-gamma	1.54E-04	IDO1, GBP1, WARS, Brca1, CDK4, Cyclin E, Caspase-3, IFN-gamma, STAT1
**45**	Transcription_Negative regulation of HIF1A function	1.65E-04	MCM3, p14ARF, HIF-prolyl hydroxylase, MCM7, ARD1, MCM2, MCM5, PRDX4, HSP70, Elongin C, EGLN1
**46**	Role of histone modifiers in progression of multiple myeloma	2.31E-04	EZH2, Tubulin alpha, HDAC2, CDK4, N-Ras, Histone H4, Caspase-3
**47**	Role of Bregs in attenuation of T and NK cells	3.12E-04	PD-1, CTLA-4, CD80, IP10, IL-2R alpha chain, Granzyme B, LAG3,

	mediated anti-tumor immune responses		IFN-gamma
**48**	Transcription_Sin3 and NuRD in transcription regulation	3.12E-04	Mi-2, SAP30, HDAC2, PSF, p66alpha, MTA2, Histone H4, Mi-2 alpha
**49**	Notch signaling in breast cancer	3.97E-04	NOTCH1 (NICD), Cyclin A, HURP, NOTCH1 receptor, Survivin, Cyclin B1, NOTCH1 (NEXT), NOTCH1 precursor, CDK2
**50**	Immune response_IFN-gamma actions on extracellular matrix and cell differentiation	4.59E-04	GCH1, TSA-1, EZH2, WARS, IP10, HDAC2, PSME2, IFN-gamma, STAT1
